# Evaluating the African arid corridor hypothesis: A meta‐analysis including the phylogenetic and biogeographical history of *Sesamothamnus*

**DOI:** 10.1002/ajb2.70192

**Published:** 2026-04-22

**Authors:** John G. Zaborsky, Jeffrey P. Rose, Ricardo Kriebel, Bryan T. Drew, Emily Moriarty Lemmon, Alan R. Lemmon, Kenneth J. Sytsma

**Affiliations:** ^1^ Department of Botany University of Wisconsin‐Madison 53706 WI USA; ^2^ Department of Plant and Agroecosystem Sciences University of Wisconsin‐Madison Madison 53706 WI USA; ^3^ Department of Evolution, Ecology, and Organismal Biology Ohio State University Columbus 43212 OH USA; ^4^ California Academy of Sciences 55 Music Concourse Drive, Golden Gate Park San Francisco 94118 CA USA; ^5^ Department of Biology University of Nebraska at Kearney Kearney 68849 NE USA; ^6^ Department of Biological Science Florida State University Tallahassee 32306 FL USA; ^7^ Department of Scientific Computing Florida State University Tallahassee 32306 FL USA

**Keywords:** African Arid Corridor, anchored hybrid enrichment, disjunct, long‐distance dispersal, migration, Pedaliaceae, vicariance

## Abstract

**Premise:**

We examined the African arid corridor (AAC) disjunction pattern of vascular plants between northeastern and southwestern Africa in the context of geological and climatic events since the late Miocene. We developed a phylogenetic and biogeographical framework for the arid‐adapted genus *Sesamothamnus* (Pedaliaceae), a classic example of the AAC disjunction pattern.

**Methods:**

A phylogenetic tree based on next‐generation sequencing of 512 nuclear genes and entire plastomes for all species of *Sesamothamnus* was time calibrated. Parsimony analyses were employed for morphological data. We gleaned the literature for examples of the AAC disjunction that were time calibrated or could be dated and compared them to climatic and geological changes from the Miocene to Pleistocene. Habit, dispersal mechanism, arid‐adapted features, and geographic source area were collected.

**Results:**

The nuclear and plastome data provide congruent phylogenies for *Sesamothamnus*, indicating that the species restricted to northeastern Africa are sister to the southwestern species and diverged about 8.4 million years ago (Ma). Time calibrated splits for the AAC disjunct pattern were obtained for 73 vascular plant clades with dates ranging from 34 Ma to the Pleistocene, with the majority dated to the late Miocene, Pliocene, and Pleistocene.

**Conclusions:**

Most clades exhibiting the AAC disjunction pattern, including *Sesamothamnus*, appear associated with several waves of aridification affecting both northeastern, eastern, and southwestern Africa involving the rise of C_4_ and CAM photosynthesis, succulence, spiny vegetation, and bovid herbivory. The AAC disjunction pattern is complex, appears to involve migration, rather than simple vicariance, and exhibits a strong southwest to northeast migration bias.

Continental Africa represents a classic biogeographical model system to examine past, present, and future distributions of its plant biota, estimated at over 65,000 species across 29 million square kilometers (Hong et al., 2021; Qian et al., 2021; Muasya et al., 2024). Despite the diversity of biomes across Africa, the continent displays a strong arid or semiarid signature (Figure 1A), with an estimated 70% of natural landscapes designated as desert, open grassland, or arid shrubland (Mayaux et al., 2004; Bobe, 2006). Due to the wide interest over the past decades in primate and hominoid evolution in Africa (e.g., Pickford, 1990; Kingston et al., 1994; Chan et al., 2019), an immense set of data has been assembled for late Tertiary and Quaternary changes in African geology, climate, and vegetation (Van Zinderen Bakker and Mercer, 1986; deMenocal, 1995; Sepulchre et al., 2006; Jacobs et al., 2010). Despite the present richness in African biodiversity and accumulated information on paleoenvironment and paleovegetation patterns, no overarching narrative appears to fully explain the origins of this biodiversity or the peculiar disjunct patterns evident today across Africa (Goldblatt, 1978; Linder, 2014; Muasya et al., 2024). Classification and subsequent assessment of biogeographical relationships among regional centers of endemism in the African flora have progressed from early subjective “phytochorological” systems (e.g., White, 1978; Figure 1B) to more objective, biota‐wide systems (e.g., Linder et al., 2012; Linder, 2014). Much of this biogeographical (and phylogenetic) research has centered on the distinctive and highly endemic flora of south and southwestern Africa and its different elements including the Cape flora and the Namaqualand semidesert flora (Goldblatt, 1978; Cowling et al., 1999; Linder, 2003, 2014; Linder and Verboom, 2015).

**Figure 1:**
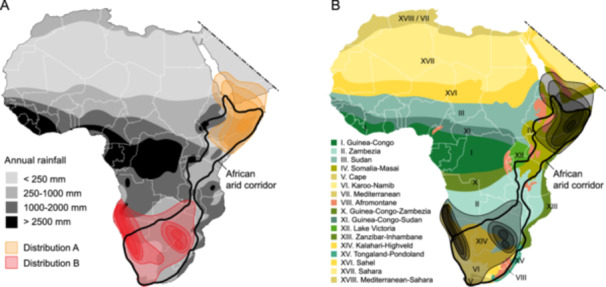
(A) Disjunct distribution of 11 representative African species or sister species (including *Sesamothamnus*) forming the basis for the African arid corridor hypothesis (modified after Jürgens, 1997). Centers of diversity of disjunct species in arid northeastern Africa and arid southwest Africa are shown in color tones relative to precipitation gradients shown in gray tones. Innermost concentric ring in each distribution represents >5 species present, next outer ring represents >4 species present, etc., and the outermost ring represents >1 species present. Rainfall gradient is modified after Wilfert et al. (2006) and Masih et al. (2014). The African arid corridor location is based on Kissling et al. (2016). (B) Centers of diversity of disjunct species in arid northeastern Africa and arid southwestern Africa (in gray tones) relative to mainland African floristic zones identified by White (1983). Floristic Zone IX (Afroalpine Archipelago Region) within the Afromontane Zone (VIII) is not shown. Comparable floristic zones in the Arabian Peninsula are based on those of Thomas (2019).

The striking and often continentwide disjunct distributional patterns seen in many African plant and animal genera (sometimes even species) have been the focus of intense biogeographical and phylogenetic research. The most complete assessment of African terrestrial biodiversity throughout the Cenozoic to the mid‐Pleistocene synthesized African climate, tectonics and 89 dated molecular phylogenetic studies (Couvreur et al., 2021). As summarized (Linder, 2014), three main disjunct distribution tracks have been proposed and variously tested: (1) the disjunction between rainforests of West and Central Africa and of the East African coastline (Faden, 1974; Couvreur et al., 2008; Dagallier et al., 2024); (2) the Afromontane disjunction of tropical montane regions linking mesic evergreen forests and grasslands across eastern Africa (White, 1978; Linder, 1994; Galley et al., 2007; Kandziora et al., 2022); and (3) the Rand Flora encompassing arid margins of the African continent (Jürgens, 1997; Sanmartín et al., 2010; Pokorny et al., 2015; Mairal et al., 2017; Rincón‐Barrado et al., 2024). The first described and most extensively studied of these disjunct patterns is the arid track within the Rand Flora, which links the arid regions of southwestern Africa (centering in Namibia) with those of northeastern Africa (centering in Somalia and around the Horn of Africa) (Engler, 1921; Balinsky, 1962; Verdcourt, 1969; de Winter, 1971; Thulin, 1994; Jürgens, 1997). The formation of these arid regions is linked to the gradual cooling and aridification of the African continent starting around 16 million years ago (Ma) in the Miocene, the subsequent montane uplift in the east and south, the reduction of the lowland rain forests in eastern Africa, the recurring expansion of semiarid woodlands, savannas, and deserts through the Pliocene–Pleistocene (Andrews and Van Couvering, 1975; deMenocal, 1995; Cerling et al., 1997; Bobe, 2006; Sepulchre et al., 2006; Feakins et al., 2013; Liddy et al., 2016).

The African arid track, commonly referred to as the African arid corridor (or AAC), has been implicated in past movements of many different plant genera and families, and even a diversity of animal lineages (Balinsky, 1962; Poynton, 1995; Herron et al., 2005; Bobe, 2006; Kissling et al., 2016; O'Connor et al., 2019; Wüster et al., 2020). The AAC likely will play an important role in future movements of biota across arid zones due to ongoing global warming (Perkins, 2020). The number of examples demonstrating the AAC pattern is not known, as a more thorough listing of its constituent taxa has not been attempted. The AAC disjunction was first mapped by Jürgens (1997). He used the distributions of 11 species or related species to explicitly demarcate the disjunct pattern from northeastern (NE) Africa to southwestern (SW) Africa (Figure 1). These disjunctions have been variously and often subjectively interpreted—via migrations through uninterrupted African arid corridors at different times in the past, via vicariance of a once more continuous arid biota, via “stepping‐stone” dispersals over short distances, and via long‐distance, continentwide dispersal events (e.g., Verdcourt 1969; Thulin, 1994; Caujape‐Castells et al., 2001; Cortes‐Burns et al., 2004; Bellstedt et al., 2012; Ali et al., 2013). The AAC demonstrates the challenges of applying modern and objective parametric models in historical biogeographical inference (Ree et al., 2005; Ree and Sanmartín, 2009; Matzke, 2014) in that information on past and present climatic and geological events, phylogenetic relationships, temporal diversification, and dispersal probabilities are all needed to clarify the mechanism(s) driving the disjunct pattern for each representative clade. As a first step in this process to elucidate the AAC and possible drivers of this disjunct pattern, we first present a meta‐analysis of published examples exhibiting the pattern by providing time splits of species and clades from NE Africa to SW Africa and examining climatic and geological associations to these times. Subsequently, we undertake a molecular and morphological analysis of one of the classic examples of the AAC disjunction used by Jürgens (1997)—the genus *Sesamothamnus* Welw. from the sesame family (Pedaliaceae R.Br.).

*Sesamothamnus* comprises six species of large shrubs native to dry and arid regions of NE and southern (S) Africa and is the sole member of the tribe Sesamothamneae Ihlenf. (Ihlenfeldt, 2002). *Sesamothamnus* exemplifies the AAC disjunct pattern (Figure 2). Two species (*S. rivae* Engl. and *S. busseanus* Engl.) are confined to NE Africa, occurring in Ethiopia, Somalia, Kenya, and Tanzania, while the remainder of the genus is disjunct in S and SW Africa. *Sesamothamnus leistneri* P. Craven ex Swanepoel & A.E. van Wyk and *S. guerichii* (Engl.) E.A. Bruce are endemic to NW Namibia. *Sesamothamnus benguellensis* Welw. is also found in NW Namibia, extending into S Angola. *Sesamothamnus lugardii* N.E. Br. ex Stapf occurs in Zimbabwe, Botswana, and NE South Africa. *Sesamothamnus* exhibits a suite of traits adapted to arid environments. The genus is unusual in the Pedaliaceae in being woody, while most of the family is herbaceous. Along with *Uncarina* Stapf. and *Pterodiscus* Hook. (tribe Pedalieae), *Sesamothamnus* is the only other genus in the family that has developed stem succulence. The four southern species have developed succulence to a greater degree than the two northeastern species (Ihlenfeldt, 2002). The genus possesses a branching system that produces long shoots with spines and short shoots that arise from the axils of these spines.

**Figure 2:**
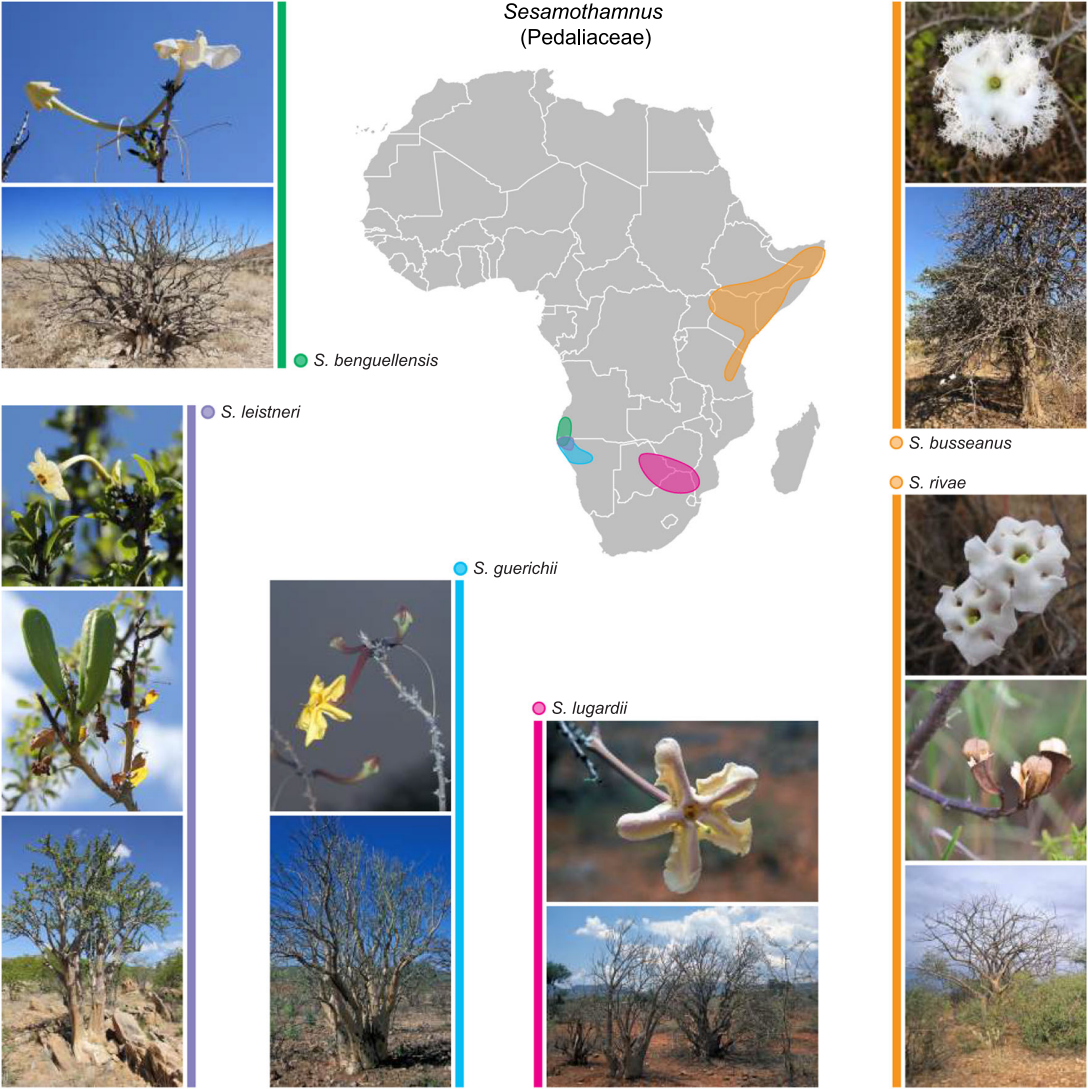
Distributional map of the six species of *Sesamothamnus* (Pedaliaceae) (modified after Ihlenfeldt and Chikuni, 1994): *S. rivae*, *S. busseanus*, *S. lugardii*, *S. leistneri*, *S. guerichii*, and *S. benguellensis*. D. Mahr provided photos of *Sesamothamnus benguellensis*, *S. guerichii*, and *S. leistneri*. S. Mahr provided photos of *S. lugardii*. D. Bell provided photos of *S. busseanus*. Photos of *S. rivae* were provided by C. Waltenberg (flower/fruit) under Wikimedia Commons and M. Olson (habit).

Refulio‐Rodriguez and Olmstead (2014) investigated Pedaliaceae in a phylogenetic context, primarily examining the placement of the family within Lamiales, while Gormley et al. (2015) investigated intrafamilial relationships. Though a few species of *Sesamothamnus* were included in these studies, no genus‐wide phylogenetic analysis is available. The family‐wide analysis (Gormley et al., 2015) included two species of *Sesamothamnus* and resolved them as monophyletic (sole genus of tribe Sesamothamneae) and sister to the tribe Sesameae (Endl.) Meisn., which is made up largely of the paraphyletic genus *Sesamum* L. (Zhigila and Muasya, 2023) but also includes *Ceratotheca* Endl., *Josephinia* Vent., and a handful of other small genera. These two tribes are sister to the tribe Pedalieae Meisn. comprising the remainder of the genera including *Uncarina* from Madagascar, a genus that radiated during the Pleistocene (Rose et al., 2025b). Thus, *Sesamothamnus* shares with its sister tribe Sesameae a suite of synapomorphic androecial features and appears to independently share stem succulence with unrelated *Uncarina* in tribe Pedalieae. However, *Sesamothamnus* also exhibits a suite of distinctive traits in the family: short lateral shoots, moth‐pollinated flowers, pollen grains in tetrads, and winged seeds from dehiscent capsules (Ihlenfeldt, 2004). Our phylogenetic study is the first to include all known taxa of *Sesamothamnus*, and we used Illumina next‐generation sequencing (NGS) to obtain sequences of over 500 nuclear genes and virtually complete plastomes from each species. This phylogenetic framework was then used to provide a time‐calibrated perspective for the diversification of *Sesamothamnus* across the continent of Africa and the formation of its AAC disjunct pattern.

## MATERIALS AND METHODS

### Phylogenetics of *Sesamothamnus*

We examined the phylogenetics and biogeography of *Sesamothamnus* in Africa by sampling 11 accessions representing all six species in *Sesamothamnus* along with four representatives from three other genera in Pedaliaceae (*Sesamum*, *Pterodiscus*, *Uncarina*) and using NGS (Table 1). *Sesamum* represents the tribe Sesameae sister to *Sesamothamnus* (Gormley et al., 2015; Rose et al., 2025b), and thus *Uncarina* and *Pterodiscus* of the tribe Pedalieae were used as a monophyletic outgroup. Silica‐dried leaves were obtained for all *Sesamothamnus* taxa; these included vouchered, living collection specimens directly collected from native habitats by Dr. Dan Mahr (Entomology Department, University of Wisconsin‐Madison, WI, USA), or from the living collection at the Huntington Botanical Garden in San Marino, CA, USA. Silica‐dried leaves of wild‐collected specimens of *Pterodiscus aurantiacus*, *Uncarina abbreviata*, and *Uncarina platycarpa* were used for DNA extraction. *Sesamum trilobum* (Bernh.) Byng & Christenh. (*Ceratotheca triloba*) was collected from the UW‐Madison Botany Department Greenhouse, WI, USA. DNA extraction used the DNeasy plant mini kit (Qiagen, Valencia, CA, USA).

**Table 1:** Species sampled for anchored hybrid enrichment sequencing of *Sesamothamnus* and outgroup genera and corresponding vouchers.

Species	Voucher
*Pterodiscus aurantiacus* Welw.	D. Mahr 2012‐28 (WIS)
*Sesamothamnus benguellensis* Welw.	D. Mahr 2012‐43 (WIS)
*Sesamothamnus busseanus* Engl.	D. Mahr s.n. (WIS)
*Sesamothamnus guerichii* (Engl.) E.A. Bruce S02	D. Mahr 2012‐44 (WIS)
*Sesamothamnus guerichii* (Engl.) E.A. Bruce S04	HBG s.n. (WIS)
*Sesamothamnus guerichii* (Engl.) E.A. Bruce S11	D. Mahr 10063 (WIS)
*Sesamothamnus guerichii* (Engl.) E.A. Bruce S12	D. Mahr 87010 (WIS)
*Sesamothamnus leistneri* P. Craven ex Swanepoel & A.E. van Wyk	D. Mahr 00045 (WIS)
*Sesamothamnus lugardii* N.E. Br. ex Stapf. S10	D. Mahr 82006 (WIS)
*Sesamothamnus lugardii* N.E. Br. ex Stapf. S14	D. Mahr s.n. (WIS)
*Sesamothamnus rivae* Engl. S07	D. Mahr s.n. (WIS)
*Sesamothamnus rivae* Engl. S08	D. Mahr 00088 (WIS)
*Sesaum trilobum* (Bernh.) E. Mey ex Hook. f.	Zaborksy s.n. (WIS)
*Uncarina abbreviata* (Baill.) Ihlenf. & Straka	Röö/Hoff No. 1167 (WIS)
*Uncarina platycarpa* Lavranos	Röö/Hoff 77/98 (WIS)

For sequencing, we utilized an anchored phylogenomics approach, anchored hybrid enrichment (AHE), conducted at the Center for Anchored Phylogenomics at Florida State University (Tallahassee, FL, USA). This method targets highly conserved “anchor” regions in the nuclear genome and samples over 500 loci including both introns and exons (Lemmon et al., 2012; Buddenhagen et al., 2016). Pedaliaceae DNAs were sequenced along with *Salvia* (Lamiaceae) DNAs (Kriebel et al., 2019; Rose et al., 2021a) and *Uncarina* (Rose et al., 2025b), and thus more in‐depth details of the methodology can be found in the cited references. Briefly, DNA was sonicated to a fragment size of between 200 and 600 bp before library preparation and indexing following a modified protocol of Meyer and Kircher (2010). Indexed samples were pooled and enriched using the Angiosperm v.1 enrichment kit (Buddenhagen et al., 2016). Sequencing was done on 4.5 PE150 Illumina HiSeq. 2500 lanes at the Translational Science Laboratory, College of Medicine, Florida State University. Read mappings, construction of consensus sequences, locus orthology assessment, and orthologous cluster alignment were detailed elsewhere (Rokyta et al., 2012; Prum et al., 2015; Buddenhagen et al., 2016; Hamilton et al., 2016; Kriebel et al., 2019; Rose et al., 2025a). Raw reads have been deposited in the NCBI Sequence Read Archive (SRA) as BioProject PRJNA1419806 and PRJNA1214378.

We assembled the nearly complete plastomes of *Sesamothamnus* and outgroup samples by mapping the off‐target reads from the AHE sequencing to a previously published plastome of *Sesamum indicum* L. (GenBank accession KCS569603). The plastomes were assembled using Geneious v.10.2.3 (Kearse et al., 2012) and the procedure detailed by Rose et al. (2021b). Raw reads were trimmed and assembled using iterative refinement of up to five times with the default Geneious mapper and medium sensitivity. Bases were called using a strict consensus approach (found in 50% or more of mapped reads). Plastome sequences were aligned using MAFFT v. 7.023b (Katoh and Standley, 2013). After alignment, ambiguously aligned or called regions were removed manually.

For the analysis of 11 samples of *Sesamothamnus* and four other species in Pedaliaceae based solely on AHE data from over 500 nuclear genes, we followed the methods of Kriebel et al. (2019) in using two phylogenetic approaches. First, the concatenated AHE data set was used to estimate a phylogeny under the GTRGAMMA model (selected with jModelTest 2; Darriba et al., 2012) implemented in RAxML v8.1.21 (Stamatakis, 2014; with default parameters), with the GTR model and the branch lengths allowed to vary across loci. One hundred bootstrap replicates were collected to estimate phylogenetic support. Second, the species tree was estimated under the coalescent model as implemented by ASTRAL‐II (v.4.9.7, Mirarab and Warnow, 2015), using bootstrapped gene trees estimated under the GTRGAMMA model in RAxML v8.1.21 (Stamatakis, 2014). Because ASTRAL should not be used for plastome genes as they represent a single linkage group (Baker et al., 2021; Doyle, 2022), the aligned cpDNA plastomes obtained from the Illumina HiSeq runs were analyzed using a Bayesian framework in MrBayes 3.2.6 (Ronquist et al., 2012) implemented in the CIPRES Science Gateway (Miller et al., 2010). Default parameters were used, set to the GTRGAMMA model, temp=0.3, and the program was run for 5,000,000 generations sampling every 10,000. As with the nuclear gene data, RAxML bootstrapping was also done with the plastome data set.

### Dating the diversification of *Sesamothamnus*

To examine temporal diversification within *Sesamothamnus*, the nuclear AHE data were used from 11 samples of *Sesamothamnus* and four outgroup species in Pedaliaceae. Because the large size of the nuclear AHE data set precluded effective Bayesian dating approaches on the entire set of loci, we utilized a subset of loci following the methods in Smith, Brown and Walker (2018a) and Rose et al. (2025b). A random set of 20 loci were sampled from all loci that contained sequences for all 15 taxa. Divergence times were estimated in BEAST 2 v.2.7.7 (Bouckaert et al., 2019), treating each locus as a separate partition, using the GTR substitution model as determined for the ML analysis, linking all trees, and implementing an optimized relaxed clock with either a yule or birth death tree prior, and selecting the yule tree prior based on log marginal likelihoods (Baele et al., 2012)—see Kriebel et al. (2019) for details. Based on the dating method for *Uncarina* (Rose et al., 2025b), three secondary‐age priors were taken from a recent study of the order Lamiales based on seven fossil constraints (Rose et al., 2022). The priors were uniformly distributed with minima and maxima based on the 95% highest posterior density (HPD) of the estimated age: the crown of Pedaliaceae (8.80–32.97 Myr), the most recent common ancestor (MRCA) of *Sesamum* and *Sesamothamnus* (2.59–18.85 Myr), and the MRCA of *Pterodiscus* and *Uncarina* (5.40–27.46 Myr). The prior for the crown of Pedaliaceae falls within the 95% CI of the estimated age for the family based on angiosperm‐wide phylogenomics (Zuntini et al., 2024). The BEAST 2 analysis was run for 100,000,000 generations (two combined runs each of 50,000,000), with sampling every 100,000 generations.

As an additional dating method for the nuclear phylogenomic data, we utilized penalized likelihood as implemented in treePL (Smith and O'Meara, 2012) on the ML or Bayesian trees. This program has been shown to estimate divergence dates similar to those with Bayesian approaches (Lagomarsino et al., 2016) and has been used when an entire phylogenomic sequence data set cannot be examined in a Bayesian framework. Based on the dates from Rose et al. (2022), the crown age of Pedaliaceae was set at 19.7 Myr, with two subsequent PL analyses using the upper and lower 95% confidence interval dates (33 and 8.8 Myr), respectively, for the crown of the family to obtain confidence intervals within *Sesamothamnus*.

### Morphological evolution in *Sesamothamnus*

Due to the great diversity of habit, vegetative, floral, and fruit features across Pedaliaceae, with many of them adaptations to arid environments, a rigorous analysis of these features awaits a more complete molecular phylogenetic framework of the entire family. However, Ihlenfeldt (2010) provided a suite of features specific to the context of morphological evolution in *Sesamothamnus*, listed the character states for each of the species, and hypothesized each of their character state transitions in the absence of any known phylogenetic framework. These explicit hypotheses involving evolution of habit, succulence, leaf indument, floral color, corolla spur, petal lobe fringing, and anther exsertion were examined using the AHE chronogram, reduced to one accession per species with the droptip function in R version 4.4.2 (R Core Team, 2024). For each of these seven characters varying in *Sesamothamnus*, the plesiomorphic state shared with genera in the sister tribe Sesameae, sister to *Sesamothamnus* (based on Gormley et al., 2015), was assigned to all outgroups. The morphological data set is available online (Appendix S1). Parsimony reconstruction used the R library phangorn (Schliep, 2011) with the function ancestral.pars. We explored both MPR and ACCTRAN character‐state optimizing options.

### Meta‐analysis of the African arid corridor hypothesis

We comprehensively surveyed the literature to locate all studies in which disjunct species, sister species, or sister clades supporting the AAC hypothesis could be identified, including from this study of *Sesamothamnus*. Species distributions were examined from taxonomic reports and monographs and from the online Global Biodiversity Information Facility (GBIF, 2025) and Plants of the World Online (POWO, 2025). Some disjunctions may overlap broadly with the AAC pattern (Figure 1) but involve SW disjunctions with more mesic, montane eastern African species for example in Asparagaceae, Campanulaceae, Convolvulaceae, Iridaceae, Juncaginaceae, Orchidaceae, and Restionaceae (Bytebier et al., 2007; Galley et al., 2007; Roquet et al., 2009; Ali et al., 2013; Bentley et al., 2014; von Mering and Kadereit, 2015; Mitchell et al., 2016), and these were not included. We used species and generic names following POWO (2025) whenever possible. We used family names following those of the Angiosperm Phylogeny Group (2016) (e.g., maintaining Wellstediaceae rather than Boraginaceae, see Vasile et al. (2025)).

This list of arid or semiarid taxa was then refined based on whether a chronogram was available in each study providing age splits of NE and SW African taxa. The chronogram for *Sesamothamnus* and outgroups generated in this study and in the analysis of *Uncarina* (Rose et al., 2025b) was additionally used to calibrate the Pedaliaceae tree in Gormley et al. (2015) to get time splits within *Pterodiscus* and *Sesamum* (including *Ceratotheca*) (Zaborsky, 2018). In some groups such as Apocynaceae (Bruyns and Klak, 2006; Thulin et al., 2008; Joubert et al., 2016; Bruyns et al., 2014, 2017), Fabaceae (Haston et al., 2005), and families within Caryophyllales (Douglas and Manos, 2007; Thulin et al., 2012; Bruyns et al., 2014b), larger supermatrix studies (Zanne et al., 2014; Fishbein et al., 2018; Smith et al., 2018b) were used to obtain age splits for taxa for which the original study had not conducted a time‐calibrated analysis. For the Cucurbitaceae, time splits from Schaefer et al. (2009) were used to obtain a PL chronogram using an undated phylogenetic tree from Thulin et al. (2018a).

For timing of the biogeographical splits, we followed the method of Galley et al. (2007) by estimating the age of the ancestral node of the branch that exhibits the change in distribution. This way of estimating ancestral node ages assumes that dispersal or vicariance closely accompanied the speciation event. Dating within‐species disjunctions (dispersal or vicariance without accompanied speciation) usually cannot be done precisely because the event could have occurred anywhere on the stem branch leading to the population sampled. Thus, this approach in using stem nodes will always estimate maximum ages of the AAC split for between‐ and within‐species examples. One other possible bias in this approach, especially for actively speciating groups, is that the phylogenetic and biogeographical signals for earlier AAC disjunctions (via dispersals or vicariance) may be blurred by more recent events (e.g., *Fagonia*, Bellstedt et al., 2012) and thus bias toward younger dates. However, this is a general problem in any biogeographical reconstruction and not restricted to this analysis. The 95% confidence intervals for age splits were obtained when available, but the results are presented for the mean dates only. However, because a large number (73) of such time splits were obtained, it is reasonable to assume that any general pattern emerging from this meta‐analysis using mean dates should be informative. The resulting timeline containing these time split dates was compared to known geological, climatic, vegetation, and faunal changes occurring across Africa during these time intervals. Whenever possible, the biogeographical pattern of the more inclusive clade containing the AAC disjunct taxa was assessed to provide insight on the likely source area for each group now exhibiting the AAC disjunct pattern: SW Africa, NE Africa, or neither (disjunct pair sister to a clade in other biogeographical areas). Finally, we summarized the adaptations of the AAC disjunct taxa to aridity, browsing pressures, and dispersibility. We obtained growth form including succulence, general type of fruit/seed dispersal unit, possession of spiny vegetative features, and either CAM or C_4_ photosynthesis pathways for each disjunct pair.

## RESULTS

### Phylogenetics of *Sesamothamnus*

**Figure 3:**
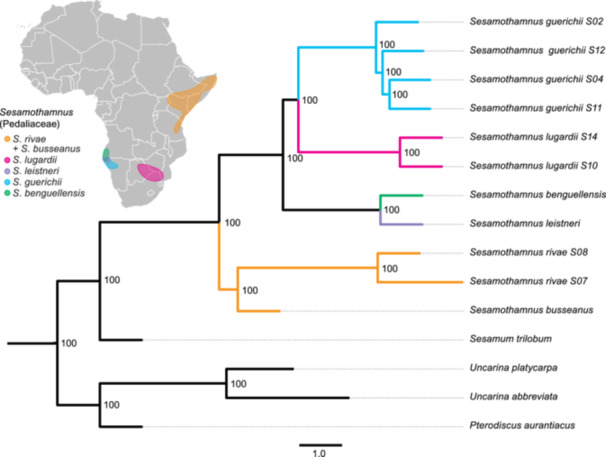
ASTRAL tree of *Sesamothamnus* and outgroup genera in the Pedaliaceae based on sequences of 512 nuclear genes obtained with anchored hybrid enrichment. The tree is in coalescent units, and bootstrap support values are indicated. The phylogentic tree shown here is topologically congruent with that based on nearly complete plastomes obtained with anchored hybrid enrichment (Appendix S2). RAxML topology based on concatenation of all nuclear genes is identical except *S. guerichii* S02 is sister to *S. guerichii* S12; bootstrap values are all 100% except for *S. guerichii* S02 + *S. guerichii* S12 (83%) and *S. guerichii* S04 + S11 (96%). The distributions of *Sesamothamnus* species are color‐coded to match placements on the tree.

The AHE sequencing provided an average of 5.6 million reads for each of the 15 samples of Pedaliaceae. The final nuclear data set included 512 loci and a concatenated length of 364,334 bp, with an average of 460 reads per locus (range: 223–867) and an average length of each locus of 725 bp (range: 597–820). Although some loci were not obtained for all accessions, an average of 470 nuclear loci (range 450–485) were sequenced with at least 250 bp each. The AHE nDNA coalescent tree of the 512 loci (Figure 3) provides strong support for both the monophyly of *Sesamothamnus* and all other relationships within the genus and the Pedaliaceae (bootstrap values of 100 for all nodes). The tree is topologically congruent to the ML tree (not shown) based on concatenation of all loci, except for the placement of one accession of *S. guerichii* within the species (ML bootstrap values provided in Figure 3). Individual species with more than one accession sampled are monophyletic. Within *Sesamothamnus*, there is a separation between the southern and northern African species in the tree, with the northern *S. busseanus* and *S. rivae* sister to a clade of the southern species. In the southern clade, the Namibian endemic, *S. leistneri* and the Angolan/Namibian *S. benguellensis*, form a clade sister to a clade of the Namibian endemic *S. guerichii* and the south‐central African *S. lugardii*.

Virtually complete plastomes were recovered from the Illumina HiSeq runs for all accessions. When aligned against the reference plastid genome of *Sesamum indicum*, the final aligned data set was 155,564 bp. The Bayesian cpDNA phylogeny (Appendix S2) is nearly identical to the RAxML tree and shows strong support for all relationships. This plastome tree depicts the same relationships as in the nDNA ASTRAL coalescent tree (Figure 3).

### Diversification and biogeography of *Sesamothamnus*

**Figure 4:**
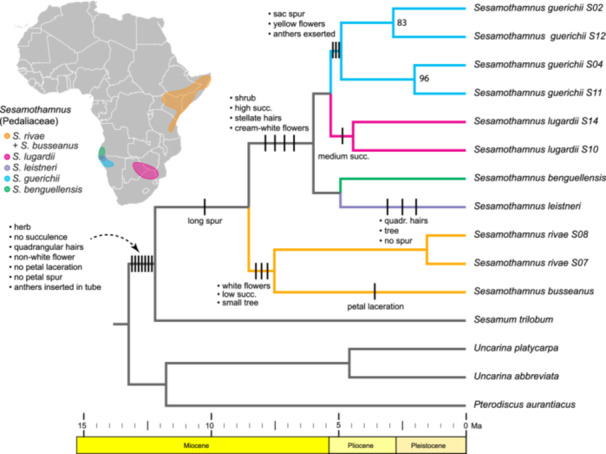
BEAST chronogram of *Sesamothamnus* and outgroup genera in the Pedaliaceae using 20 nuclear genes based on anchored hybrid enrichment Illumina sequencing (95% CI dates for all branch nodes provided in Appendix S3). The distributions of *Sesamothamnus* species are color‐coded for northeastern versus southwestern Africa in the African arid corridor (see Figure 1). Because the four outgroup species are only representatives of larger, biogeographically complex clades across Africa, Madagascar, and the Indian Ocean arc, they are not color‐coded for area. Ancestral character reconstruction of vegetative and floral characters (Appendix S1) is depicted using parsimony (Appendix S5).

The BEAST chronogram (Figure 4; 95% CI provided in Appendix S3) shows the crown radiation of *Sesamothamnus* in the late‐Miocene at 8.4 Ma, and thus the time split between the NE and SW African clades. The separation between the two NE species, *S. rivae* and *S. busseanus*, occurred soon after (7.4 Ma) indicating a long period of existence of the genus in NE Africa. The four species in SW Africa diversified just before the Pliocene at 5.5 Ma, but with the three species restricted to the extreme arid region of northern Namibia and southern Angola not forming a clade. The more southwestern *S. guerichii* is sister to *S. lugardii*, which is disjunct in south‐central Africa, and this Pliocene split occurred soon after the SW African clade origin (4.7 Ma). Similarly, the two more northerly species at the border of Namibia and Angola, *S. benguellensis* and *S. leistneri*, split in the Pliocene (4.6 Ma). TreePL dating (Appendix S4) gave an older crown radiation of *Sesamothamnus* (12.8 Ma) but within the 95% CI.

### Trait evolution in *Sesamothamnus*

The plesiomorphic suite of traits at the stem of *Sesamothamnus*, based on distributions of character states across its sister tribe Sesameae and across most of the tribe Pedalieae, is inferred to include herbs (occasionally woody‐based), no succulence, mucilaginous hairs with quadrangular‐shaped heads, non‐white‐colored flowers, no petal fringe or spur, and anthers inserted into the corolla throat (Figure 4; see parsimony reconstruction of each trait in Appendix S5). Before the crown radiation of *Sesamothamnus* in the late‐Miocene (stem lineage 12.0–8.4 Ma), the clade evolved some degree of woody stem succulence and white (or cream) flowers with a corolla spur. The NE African clade evolved the small tree habit with low succulence and white flowers early on with a subsequent shift to petal fringing in *S. busseanus*. The SW African stem clade evolved extreme stem succulence in their shrubby habit, dense mucilaginous leaf hairs with stellate heads, and cream‐white flowers. The high succulence was retained in NW Namibia and SW Angola but was reduced in *S. lugardii* located farther southeast. *Sesamothamnus guerichii* also acquired three unique floral features: switch to yellow flowers, sac‐like spurs, and exserted anthers. Similarly, *S. leistneri* acquired a more tree‐like habit, reversal to quadrangular shaped hairs, and loss of the petal spur.

### Meta‐analysis of the African arid corridor hypothesis

This survey covered 73 AAC plant examples with dated biogeographical splits (Table 2). A number of other known or suspected NE–SW disjunctions are not listed here because lack of sampling or unresolved relationships precluded their use at this time (e.g., *Ceropegia* sects. *Huernia*, *Orbea* [Bruyns et al., 2017], *Boerhavia* [Smith et al., 2018b], some *Euphorbia* [Bruyns et al., 2011] in Euphorbiaceae; *Limeum* in Limeaceae [Jeffrey, 1961]; *Chorisochora* and other genera in Acanthaceae [Vollesen, 1994]; Scrophulariaceae [Villaverde et al., 2023]). These 73 AAC examples include 32 families and 60 genera. The disjuncts are widely spread across monocots (4), rosids (31), caryophyllids (12), and asterids (26). Cucurbitaceae (9) has the largest number of disjuncts, followed by Apocynaceae (8), Asteraceae (6), Euphorbiaceae (4), Pedaliaceae (4), and Zygophyllaceae (4). The biogeographical origin of the more inclusive clades giving rise to these AAC disjunctions is more often SW Africa (Table 2). Of the 73 disjunctions, 54 origins could be confidently placed in either SW or NE Africa, and 34 (63%) of these 54 arose out of a SW African clade.

**Table 2:** Seventy‐three plant taxa exhibiting the northeastern (NE) Africa–southwestern (SW) Africa disjunction pattern of the African arid corridor hypothesis. Family names follow those of APG IV (Angiosperm Phylogeny Group, 2016). Species names follow POWO (2025) with previous names in parentheses. Taxa/clades names in bold are SW African. Mean age splits of the disjunction from dated chronograms of cited reference with 95% confidence interval if provided; last reference used to obtain date or a secondary date to construct a PL chronogram. *Asterisks denote single species disjunct between SW and NE Africa with age of stem node with sister species. The biogeographical region of origin within Africa (NE, SW) of the more inclusive group is provided; NE‐SW is listed if the larger inclusive group is outside this disjunct area or is uncertain. Growth form, seed/fruit dispersal type, presence of spines, and arid‐adapted photosynthetic pathways are provided for each disjunct pair.

Family	Taxon	Age (Myr)	Origin	Habit	Dispersal type	Spinescence	C_4_/CAM	Reference
Acanthaceae	***Blepharis grossa*** – *B. kuriensis* clade	1.6 (2.0–0.97)	SW	annual herb	Capsule, gravity	spiny	C_4_	Fisher et al. (2015); Stata et al. (2025)
Aizoaceae	*Aizoon canariense	4.9	SW	succulent herb	capsule, hydrochory	none	none	Thulin et al. (2012); Smith et al. (2018b)
Aizoaceae	***Delosperma*** **(** ***Ectotropis*** **)** ***alpina** – D. harazianum*	1.5 (2.8–0.5)	NE	succulent herb	hygrochastic capsule	none	CAM	Valente et al. (2014); Liede‐Schumann & Newton (2018);
Aizoaceae	***Tribulocarpus dimorphanthus** ‐ T. retusus*	4.2	SW	shrub	spiny to winged fruit,/epizoochory or wind	none	none	Thulin et al. (2012); Smith et al. (2018b)
Anacampsero‐taceae	***Anacampseros*** SW clade ‐ *Anacampseros* NE clade	1.3(5.6–0)	SW	succulent herbs	capsule, gravity	none	CAM	Thulin (2002); Vásquez‐Cruz et al. (2025)
Apocynaceae	***Adenium oleifolium*** **SW clade** ‐ *Adenium somalense* NE clade	4.6 (6.0–3.5)	NE‐SW	shrubs/trees	capsule, arillate seed	none	none	Dimmitt & Edwards (2021) (pers. comm.)
Apocynaceae	***Ceropegia*** **SW clade** ‐ *Ceropegia crassa*	7.6	NE	stem succulent	follicle, wind	none	CAM	Bruyns et al. (2017); Fishbein et al. (2018)
Apocynaceae	***Cibirhiza albersiana** ‐ C. dhofarensis*	6.5	NE	woody geophyte with perennial vines	follicle, wind	none	none	Thulin et al. (2008); Fishbein et al. (2018)
Apocynaceae	***Cryptolepis capensis*** **clade** ‐ *C. nuguulensis* clade	5.1	NE	vine to shrublet	follicle, wind	none	none	Joubert et al. (2016); Fishbein et al. (2018)
Apocynaceae	***Duvalia angustiloba** ‐ D. eilensis*	2.6	NE‐SW	stem succulent	follicle, wind	none	CAM	Bruyns et al. (2017); Fishbein et al. (2018)
Apocynaceae	***Fockea** ‐ Cibirhiza*	6.5	NE	woody geophyte with perennial vines	follicle, wind	none	none	Bruyns & Klak (2006); Thulin et al. (2008); Fishbein et al. (2018)
Apocynaceae	***Huernia kennedyana** ‐ H. keniensis*	0.5	SW	stem succulent	follicle, wind	none	CAM	Bruyns et al. (2014a); Fishbein et al. (2018)
Apocynaceae	***Leptadenia (Orthanthera)*** **SW clade** ‐ *Leptadenia* NE clade	5.3	SW	shrub	follicle, wind	none	none	Meve et al. (2016); Fishbein et al. (2018)
Asphodelaceae	***Aloe*** **SW clade** ‐ *Aloe* NE clade	10.0	SW	succulent herb	Capsule, gravity	none	CAM	Grace et al. (2015)
Asteraceae	***Blumea*** **(** ***Doellia*** **)** ***afra** – B. (D.) bovei*	1.6 (2.9–0.8)	NE	herb	plumose achene	none	none	Nylinder et al. (2016)
Asteraceae	***Euryops*** **SW clade** ‐ *E. prostratus* clade	4.3 (7. –2.5)	SW	shrub	achene	none	none	Devos et al. (2010)
Asteraceae	***Geigeria spinosa** ‐ G. alata*	1.9 (3.7–0.8)	SW	herb	plumose achene	prickly leaves	none	Nylinder et al. (2016)
Asteraceae	***Nicolasia costata** ‐ N. pedunculata*	0.3 (1.0–0.1)	SW	herb	plumose achene	none	none	Nylinder et al. (2016)
Asteraceae	***Pegolettia pinnatilobata** ‐ P. senegalensis*	0.8 (1.9–04)	SW	herb	plumose achene	none	none	Nylinder et al. (2016)
Asteraceae	***Sphaeranthus*** **SW clade** ‐ *Sphaeranthus* NE clade	3.3 (5.1–2.0)	NE‐SW	herb	achene	none	none	Nylinder et al. (2016)
Burseraceae	***Commiphora pyracanthoides** ‐ C. kua* (*habessinica*)	2.7	NE	tall shrub	drupe	spiny	none	Weeks & Simpson (2007); Smith & Brown (2018)
Burseraceae	*Commiphora schimperi	6.6	SW	tall shrub	drupe	spiny	none	Weeks & Simpson (2007); Smith & Brown (2018)
Caprifoliaceae	***Scabiosa africana*** **SW clade** ‐ *S. austroafricana*	1.0 (1.8–0.4)	SW	herb	awned achene	none	none	Carlson et al. (2012)
Colchicaceae	***Colchicum asteroides*** **SW clade** ‐ *C. schimperianum*	9.6 (12–5)	SW	herb	capsule, gravity	none	none	Caujape‐Castells et al. (2001); Chacón & Renner (2014)
Crassulaceae	***Cotyledon orbiculata** ‐ C. barbeyi*	4.1 (6.0–2.0)	SW	succulent herb	follicle, gravity	none	CAM	Bruyns et al. (2019)
Crassulaceae	***Crassula*** **SW clade** ‐ *C. globularioides, C. alba*	3.0 (6.0–2.0)	SW	succulent herb	follicle, gravity	none	CAM	Bruyns et al. (2019)
Crassulaceae	***Crassula expansa** ‐ C. volkensii*	4.8 (6.5–2.5)	SW	succulent herb	follicle, gravity	none	CAM	Bruyns et al. (2019)
Cucurbitaceae	***Coccinia rehmannii** ‐ C. microphylla*	3.2 (5.1–1.5)	NE	perennial vine	berry	none	none	Holstein & Renner (2011)
Cucurbitaceae	***Cucumis asper** ‐ Oreosyce africana* (*C. subsericeus*)	4.3	NE	herbaceous vine	berry	none	none	Schaefer et al. (2009); Thulin et al. (2018a)
Cucurbitaceae	***Cucumis bryoniifolius** ‐ C. kelleri*	4.6	NE‐SW	herbaceous vine	berry	none	none	Schaefer et al. (2009); Thulin et al. (2018a)
Cucurbitaceae	***Cucumis meeusei** ‐ C. carolinus*	3.8	NE‐SW	herbaceous vine	berry	none	none	Schaefer et al. (2009); Thulin et al. (2018a)
Cucurbitaceae	***Cucumis rigidus** ‐ C. baladensis*	2.3	NE‐SW	herbaceous vine	berry	none	none	Schaefer et al. (2009); Thulin et al. (2018a)
Cucurbitaceae	***Cucumis zambianus** ‐ C. insignis*	1.6	NE	herbaceous vine	berry	none	none	Schaefer et al. (2009); Thulin et al. (2018a)
Cucurbitaceae	***Cucumis zeyheri** ‐ C. messorius*	1.7	SW	herbaceous vine	berry	none	none	Schaefer et al. (2009); Thulin et al. (2018a)
Cucurbitaceae	***Dactyliandra welwitschii** ‐ Trochomeria nigrescens (D. nigrescens)*	7.0 (9.5–4.2)	NE‐SW	herbaceous vine	berry	none	none	Lindner et al. (2017)
Cucurbitaceae	***Trochomeria debilis** ‐ T. stefaninii (Dactyliandra stefaninii)*	2.6 (4.3–1.0)	SW	herbaceous vine	berry	none	none	Lindner et al. (2017)
Didiereaceae	***Portulacaria*** **(** ***Ceraria*** **) SW clade**, *Calyptrotheca*	5.5	NE‐SW	shrub, tree	indehiscent with aril	none	CAM	Bruyns et al. (2014b); Smith et al. (2018b)
Euphorbiaceae	***Euphorbia*** **sect.** ***Anthacanthae*** ‐ *E*. sect. *Balsamis*	21.0	NE	stem succulent	capsule, gravity	spiny	CAM	Bruyns et al. (2011); Peirson et al. (2013)
Euphorbiaceae	***Euphorbia genistoides** ‐ E. schimperiana*	19.5	NE‐SW	herb to shrub	capsule, gravity	none	none	Bruyns et al. (2011); Peirson et al. (2013)
Euphorbiaceae	***Euphorbia mauritanica** ‐ E. schimperi*	6.5	SW	stem succulent	capsule, gravity	none	CAM	Bruyns et al. (2011); Peirson et al. (2013)
Euphorbiaceae	*Euphorbia* sect. *Tirucalli*	3.2 (4.9–1.7)	NE‐SW	shrub	capsule, gravity	none	CAM	Dorsey et al. (2013); Horn et al. (2014)
Fabaceae	***Indigofera nebrowniana** ‐ I. eremophila* NE clade	3.0	NE	shrublet to shrub	legume	none	none	Schrire et al. (2009)
Fabaceae	*Indigofera trigonelloides	5.7	NE‐SW	shrublet to shrub	legume	none	none	Du Preez et al. (2025)
Fabaceae	***Parkinsonia africana*** ‐ *P. sciona* NE clade	4.0	NE‐SW	shrub	legume, gravity	spiny	none	Haston et al. (2005); Zanne et al. (2014)
Geraniaceae	*Monsonia angustifolia	0.9 (1.1–0.3)	SW	annual herb	barbellate seed	none	CAM	García‐Aloy et al. (2017)
Geraniaceae	***Monsonia drudeana*** **SW clade** ‐ *M. longipes* NE clade	14.9 (23–12)	SW	suffrutescent herb	plumose & barbellate seed	none	CAM	García‐Aloy et al. (2017)
Geraniaceae	*Monsonia glauca	1.5 (3.9‐–0.9)	NE	suffrutescent herb	plumose seed	none	CAM	García‐Aloy et al. (2017)
Gisekiaceae	***Gisekia africana*** **SW clade** ‐ *G. diffusa* NE clade	6.4 (11.0–2.6)	NE	annual to perennial herb	winged mericarp	none	C_4_	Bissinger et al. (2014)
Gisekiaceae	***Gisekia paniculata*** **SW clade** ‐ *G. haudica* NE clade	9.4 (16.3–4.7)	SW	annual to perennial herb	winged mericarp	none	C_4_	Bissinger et al. (2014)
Kewaceae	*Kewa bowkeriana	1.8 (4.5–1.5)	SW	semi‐succulent herb	capsule, gravity	none	none	Thulin et al. (2018b)
Lamiaceae	***Salvia*** **SW clade** ‐ *S. nilotica*	5.0 (11.6–1.6)	NE‐SW	perennial herb	nutlet	none	none	Kriebel et al. (2019)
Lamiaceae	***Salvia aurita** ‐ S. somalensis*	4.2 (5.3–0.7)	SW	perennial herb	nutlet	none	none	Kriebel et al. (2019)
Limeaceae	***Limeum arabicum** ‐ L. pterocarpum*	18.8	SW	subshrub	schizocarp	none	none	Smith et al. (2018b)
Loasaceae	***Kissenia capensis** ‐ K. arabica*	3.9 (7.5–2.0)	NE‐SW	subshrub	capsule, gravity	bristly hairs	none	Castillo et al. (2019)
Moringaceae	***Moringa ovalifolia** ‐ M. rivae* NE clade	14.7(20.1–9.8)	NE	shrub, tree, bottle tree	capsule, wind	none	none	Carvalho & Renner (2012)
Neuradaceae	***Grielum** ‐ Neurada*	25.5 (52–6.7)	SW	annual, perennial herb	indehiscent, epizoochory or wind	none	none	Hernández‐Gutiérrez & Magallón (2019)
Nyctaginaceae	***Commicarpus squarrosus*** **SW clade** ‐ *Commicarpus* NE clade	2.8	NE	scrambling subshrub	glandular achene	none	none	Douglas & Manos (2007); Struwig & Siebert (2013); Smith et al. (2018b)
Pedaliaceae	***Pterodiscus aurantiacus** ‐ Pterodiscus* NE clade	3.8 (6.2–0.5)	NE	caudiciform with herbaceous stems	capsule, wind	none	none	Gormley et al., 2015; Zaborsky et al. this paper
Pedaliaceae	***Sesamothamnus*** SW clade ‐ *Sesamothamnus* NE clade	8.4 (13.9–3.9)	NE‐SW	shrub	capsule, winged seeds	petiole spiny	none	Zaborsky et al., this paper
Pedaliaceae	***Sesamum trilobum*** **(** ***Ceratotheca triloba** ‐ S. sesamoides* [*C. sesamoides*])	4.2 (6.3–2.1)	NE‐SW	herb	capsule, gravity	none	none	Gormley et al., 2015; Zaborsky et al. this paper
Pedaliaceae	***Sesamum*** SW clade ‐ *S. alatum*	2.2 (3.7–0.3)	SW	herb	capsule, gravity	none	none	Gormley et al., 2015; Zaborsky et al. this paper
Poaceae	***Stipagrostis*** SW clade ‐ *Stipagrostis* NE clade	6.8 (9.1–4.5)	SW	graminioid	caryopsis	none	C_4_	Cerros‐Tlatilpa et al. (2011)
Poaceae	***Trichoneura schlechteri ‐** T. ciliata*	1.8 (2.2– 0.8)	SW	graminioid	caryopsis	none	C_4_	Oulo et al. (2025)
Portulacaceae	*Portulaca wightiana	7.6	NE‐SW	succulent herbs	capsule, gravity	none	CAM	Gilbert & Phillips (2000); Ocampo & Columbus (2012)
Rubiaceae	***Plocama crocyllis*** ‐ *Plocama* “*yemenensis* group”	5.2 (8.5–2.6)	NE	tree/large shrub	drupe	none	none	Rincón‐Barrado et al. (2021)
Rutaceae	***Thamnosma africana*** SW clade ‐ *T. socrotana* NE clade	8.5 (12.1–5.3)	NE‐SW	woody herb, subshrub	capsule, gravity	none	none	Thiv et al. (2011)
Scrophulariaceae	***Camptoloma rotundifolium** ‐ C. lyperiiflorum*	4.3 (5.9–1.9)	SW	perennial or annual herb	capsule,?	none	none	Culshaw et al. (2021)
Tamaricaceae	***Tamarix usneoides*** ‐ *T. aphylla*	5.8 (10.5–2.1)	NE	shrub	capsule, wind	none	none	Terrones & Juan (2023)
Wellstediaceae (Boraginaceae)	***Wellstedia dinteri** ‐ Wellstedia* NE clade	34.0 (49.2–21.2)	NE‐SW	shrub	capsule, gravity	petiole spiny	none	Luebert et al. (2016)
Zygophyllaceae	***Fagonia rangei*** and ***F. minutistipula** ‐ Fagonia* NE clade	4.3 (6.5–2.2)	NE	woody herb, shrub	capsule, gravity	spiny	none	Wu et al. (2018)
Zygophyllaceae	***Zygophyllum patenticaule*** SW clade ‐ *Z. decumbens*	4.7 (7.3–1.3)	SW	shrub	capsule, gravity or wind	none	C_4_	Bellstedt et al (2012); Wu et al. (2015), 2018
Zygophyllaceae	***Zygophyllum rigidum*** SW clade ‐ *Z. coccineum* NE clade	5.3 (8.6–2.4)	SW	shrub	capsule, gravity or wind	spiny	C_4_	Bellstedt et al (2012); Wu et al. (2015), 2018
Zygophyllaceae	*Zygophyllum simplex	3.9 (5.6–1.2)	SW	shrub	capsule, gravity or wind	none	C_4_	Bellstedt et al (2012); Wu et al. (2015), 2018

**Figure 5:**
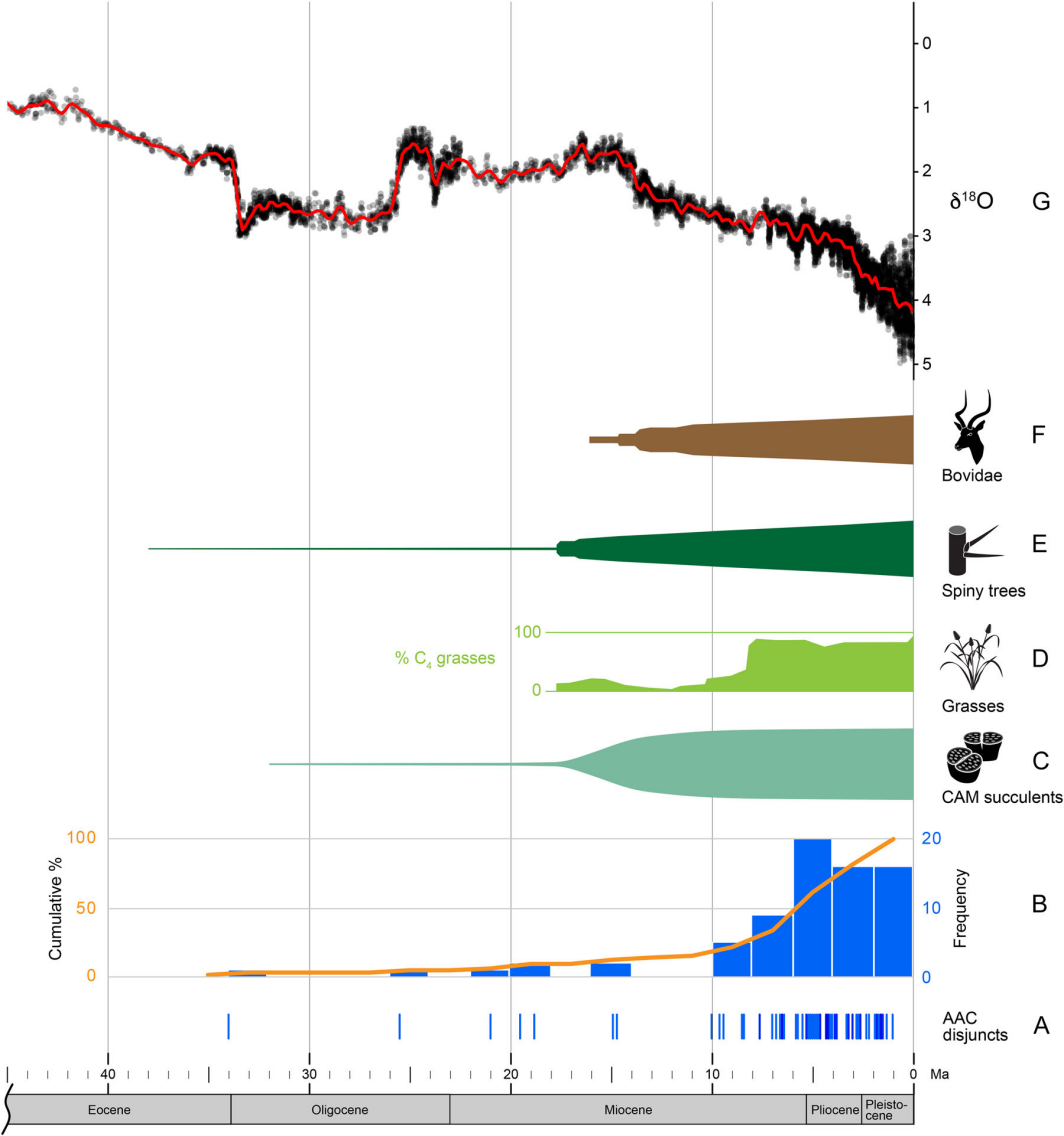
(A) Timeline of 73 time‐calibrated disjunct species, species pairs, or clades between northeastern and southwestern Africa exemplifying the African arid corridor. Intensity of blue bars increases when ages of different disjuncts overlap. (B) Histogram of the 73 time‐splits in 2‐Myr bins showing cumulative percentage of events from the Eocene to Pleistocene. (C) Diversification of CAM succulent lineages in Africa using the rapid diversification within the core Ruschiodeae in Aizoaceae (Klak et al., 2004; Arakaki et al., 2011) as exemplary. Fossil record evidence in Africa for relative abundance of (D) C_4_ grasses, (E) spiny trees, and (F) bovid fauna indicative of arid conditions across eastern Africa (graphs based on Charles‐Dominique et al., 2016). (G) Temporal variation of the oxygen isotope ratio (δ^18^O relative to δ^16^O) in shells of foraminifera from near‐global compilation of ocean sediment cores serving as an inverse proxy for global temperature decline in the Tertiary (Zachos et al., 2001; Marcot et al., 2016). Gray dots are 5‐point running means of the original raw data, and the red trend line was obtained by using a generalized additive model and smoothed with *K* = 180.

A timeline and histogram of the AAC time splits for the 73 disjuncts are shown in Figure 5A, B. Two thirds of the disjunctions (50, 68%) are restricted to the Pliocene and Pleistocene. All but three of the remainder (21, 29%) are Miocene in age, with the majority of these near the Miocene–Pliocene boundary. The oldest two disjunctions are Oligocene in age. The first involves disjunctions within the monogeneric family Wellstediaceae (*Wellstedia)* at 34.0 Ma. The second (25.5 Ma) occurs in the small, woody herbaceous to shrubby family Neuradaceae and marks the divergence of the two SW African genera, *Grielum* and *Neuradopsis*, from the NE (and N) African *Neurada*. There are eight examples of individual species exhibiting the AAC disjunct pattern. Of the six species examples with resolved biogeographical ranges, five originated in the SW region. Acknowledging the issue that many of these dates are maximal ages relative to sister species and thus biased, they average 4.1 Ma (range 0.9–7.5 Ma). Intrageneric disjunctions (60 examples) average 5.9 Ma (range 0.3–34 Ma). Intergeneric disjuncts (acknowledging that generic circumscriptions are problematic or changing for some of these groups, e.g., Apocynaceae) are considerably less common (5) and show an average of 9.8 Ma separation (range 4.3‐25.5 Ma).

The AAC disjuncts display a wide variety of growth forms (Table 2): woody‐based and succulent herbs, graminoids, herbaceous and woody vines, and woody and succulent subshrubs, shrubs, and trees. Annual or perennial herbs, often with a woody caudex or succulence, dominate with 40 (55%) species falling in this category. Herbaceous or woody vines are common with 12 (16%) species. Subshrubs or shrubs include 16 (22%) species, while trees including succulent or bottle stems are less common with three (4%) species. Graminoids are represented by only two species. Species possessing dehiscent fruits (capsules, follicles, legumes, schizocarps) are most common and seen in 39 disjunct cases (53%) (Table 2). Less common (21 species, 29%) are indehiscent, single‐seeded dry fruits, mostly achenes or caryopses, sometimes plumose, glandular, or spiny. Fleshy fruits (berry, drupe, pepo, arillates) occur in only 13 (18%) of the species and are restricted to Cucurbitaceae, Rubiaceae, and Didiereaceae. The timing of the large majority of AAC disjunctions are associated with the rise of arid adapted CAM succulence (Figure 5C) and C_4_ (Figure 5D) photosynthesis, spiny vegetation (Figure 5E), growth forms in response to bovid herbivore pressure (Figure 5F), and associated global temperature decline (Figure 5G), all starting in the mid‐Miocene and increasing into the Pliocene and Pleistocene. The 73 disjunction pairs include CAM (17 pairs), C_4_ (8 species), succulent (12 pairs), and spiny (11 pairs) plants.

## DISCUSSION

### *Sesamothamnus*, a classic but old example of the AAC disjunction

*Sesamothamnus* has long been considered one of the classic AAC disjunction examples due to the restricted distribution of its species to the areas of endemism in NE and SW Africa (Figures 1, 2). Its distinctive disjunction was noted by early workers on the African flora (Verdcourt, 1969; de Winter, 1971; Goldblatt, 1978; Jürgens, 1997) and featured prominently in the first distribution map of the disjunction (Jürgens, 1997). The AHE data for nuclear single‐copy genes and the plastid genome provide strong and consistent relationships within *Sesamothamnus*. Thus, the emerging phylogenetic framework for *Sesamothamnus* offers detailed insight into the relationships, temporal diversification, and biogeography of this African genus.

The phylogenetic results along with the dating analysis indicate that the crown radiation of the genus at 8.4 Ma in the late Miocene marks the NE–SW African disjunction for *Sesamothamnus*, the 12th‐oldest AAC disjunction. Due to the widespread biogeographical distributions of many of its closest genera, the ancestral area for *Sesamothamnus* (either NE or SW or both) cannot presently be determined with this data (see Figure 4); however, a SW origin is plausible based on the finding that the family has a likely southern Africa origin (Zhigila and Muasya, 2023). *Sesamothamnus* thus evolved during the start of the aridification in Africa and the rise of arid‐adapted features in its vegetation and associated fauna (Figure 5). The genus comprises woody shrubs or small trees, possessing flowers with a sphingophilous syndrome of pale corollas that open in the evening and release a strong scent (Johnson and Raguso, 2016) and disperse via capsules that release winged seeds (Ihlenfeldt, 2004, 2010). The northern species of *Sesamothamnus*, *S. busseanus* and *S. rivae*, are both distributed throughout Kenya, Tanzania, Somalia, and Ethiopia (Ihlenfeldt, 2004). These two species occupy bushland and grassy woodlands, usually on rocky soils, throughout their ranges and differ from the southern species in having less swollen and succulent stems. The two species differ from each other most markedly in their corolla lobes: The lobes are fringed (except the basal one) in *S. busseanus*, while in *S. rivae* all the lobes have entire margins (Figure 4). Both species share leaves that are only sparsely glandular on the adaxial surfaces with the plesiomorphic mucilaginous hairs with quadrangular shaped heads, white flowers, and slender nectar spurs. These two species have always been considered closely related due to these vegetative and floral similarities and their shared distribution (Ihlenfeldt, 2010).

The southern clade is split into two smaller clades, one containing the Namibian endemic *S. guerichii* and the south‐central African *S. lugardii*; the ranges of these two species do not overlap (Figure 2). *Sesamothamnus guerichii* has the largest range of any of the Namibian species. The flowers of these two species are quite different, with the corollas of *S. lugardii* having a short nectar spur (to 1.5 cm), while those of *S. guerichii* have lost the ancestral feature of nectar spurs and possess only a short sac‐like protrusion at the base. *Sesamothamnus guerichii* is also unique in possessing yellow flowers. The anthers distinctly protrude from the mouth of the corolla tube in *S. guerichii*, while in *S. lugardii* they are included. Both species are similar in overall appearance, being large, spiny shrubs to 5 m with swollen branches. They also share leaves that are similar in size and shape and whose surfaces are densely mucilaginous with hairs bearing stellate‐shaped heads. The second southern clade comprises two species, *S. leistneri* and *S. benguellensis*. The recently described *S. leistneri* from the Kaokoveld in Namibia (Swanepoel and Van Wyk, 2023) differs from its sister species *S. benguellensis* and the sympatric *S. guerichii*, in having a more treelike growth form with branches forming higher up on the trunk rather than near the base and in possessing peeling bark (Mannheimer and Curtis, 2009; Swanepoel and Van Wyk, 2023). In addition to the listed vegetative characters, *S. leistneri* has corolla tubes that lack nectar spurs (the second independent loss in the genus), while *S. benguellensis* has nectar spurs to 3.7 mm long.

The relationships we recovered in the genus do not match the hypotheses put forward by Ihlenfeldt (2010). He argued that it would be difficult to uncover phylogenetic relationships due to the high similarity of multiple characters among the taxa. Indeed, without flowers, many of the species are difficult to tell apart. Ihlenfeldt (2010) argued that the lack of a nectar spur was a primitive character state and that the sac‐like protrusion was an intermediate between the two extremes. Our analyses show that the possession of a nectar spur most likely has been lost twice: in *S. leistneri* and *S. guerichii* (Figure 4; Appendix S5). *Sesamothamnus guerichii* has only a small sac‐like protrusion at the base of the corolla tube, while *S. leistneri* lacks a spur altogether. These two taxa are not closely related, despite having completely sympatric ranges. The phylogenetic relationships uncovered and the reconstruction of morphological characters suggest that these two species have converged upon a short nectar spur for some specific pollinator, rather than this being a primitive character state as suggested by Ihlenfeldt (2010). *Sesamothamnus guerichii* is also unique in having yellow flowers, while all other species have white to cream‐white flowers.

*Sesamothamnus leistneri* presents an interesting puzzle in the genus. Ihlenfeldt (2010) speculated that this species was related to the NE African species (*S. busseanus* and *S. rivae*) because it possesses what he considered to be primitive character states: large, treelike growth form, spurless flowers, sparse covering of mucilage glands on the leaves, and a “relict” distribution. The AHE and plastome data, however, clearly indicate that *S. leistneri* and SW African *S. benguellensis* are sister species (Figures 3, 4; Appendix S2). Most of the suite of character states that Ihlenfeldt (2010) considered primitive are now shown to be derived (Figure 4). Additionally, *S. leistneri* possesses a unique flowering strategy during the rainy season, when leaves are fully developed, whereas all other species flower before the onset of the rainy season and when they are completely leafless.

### Climatic, geological, and paleobotanical setting of the African arid corridor

The present aridity of much of Africa outside the equatorial forests and moist woodlands (Figure 1; Pokorny et al., 2015), especially as it is expressed generally in the Rand Flora (Sanmartín et al., 2010; Rincón‐Barrado et al., 2024) and more strikingly in the AAC disjunction pattern, is in stark contrast to the more widespread mesic and wet conditions seen during the Eocene, Oligocene, and Early Miocene, especially in eastern Africa, as evidenced by plant and animal fossils (Andrews and VanCouvering, 1975; Bobe, 2006). Following the Eocene peak in global temperatures about 50 Ma (Figure 5G; Zachos et al., 2001), declines in both temperature and rainfall accelerated the expansion of more open woodland, grassland, and arid habitats worldwide and notably in Africa. This expansion of open habits, along with its arid‐adapted vegetation and associated cursorial fauna, however, is one characterized by a complex interaction of climatic, geological, and biotic factors resulting in multiple biome shifts and reversals.

Eastern Africa is key to the AAC hypothesis because its geological and climatic evolution dictates the presence and timing of arid corridors for direct overland migration of plant and animal species and because mesic barriers prevent any such exchange. Specifically, the widening and narrowing of the proposed arid track running between Kenya and Zambia/Malawi (Figure 1), the “Kenya‐Malawi gap” of Jürgens (1997), is critical to the AAC hypothesis. Likewise, the rise of arid poles for centers of plant diversification in both SW and NE Africa sets the timing for possible starts of AAC disjunctions now present between the two arid regions. The role of variation in the major Milankovitch events over the last 660,000 years causing these alternating climatic changes in eastern Africa is well documented (Duesing et al., 2021). Wet–arid changes in the eastern African climate were mainly caused by changes in orbital eccentricity, with relatively dry but variable climates occurring during orbital eccentricity minima, and increased precipitation, interspersed with distinctly dryer phases associated with orbital eccentricity maxima. Additionally, it has been demonstrated that during this same time interval, opposing arid and wet conditions existed between eastern and western Africa: During La Niña, eastern Africa experienced drier conditions than western Africa and vice versa during El Niño events (Kaboth‐Bahr et al., 2021).

Paleobotanical evidence for mesic forests and woodlands in Ethiopia, Kenya, and Uganda is seen during the Early Miocene (Bobe, 2006); similarly, the Early Miocene mammalian faunas of east Africa had a distinctive forest character (Cerling et al., 1997). Climate and vegetation modeling further supports the presence of forest biomes and warmer, more humid conditions in east Africa during the first half of the Miocene (Henrot et al., 2017). The Early Miocene rise of the great stratovolcanoes in eastern Africa and their substantial increase in the Middle Miocene (15 Ma) likely initiated the first montane forests on their slopes (Andrews and VanCouvering, 1975). However, dry seasonal temperate legume woodlands also existed in eastern Africa during the Early Miocene and their presence precludes continuous mesic forest throughout eastern equatorial Africa (Bonnefille, 2010). Indeed, fossil sites indicate the existence of a mosaic of mesic and seasonal forests as well as C_3_‐grass‐dominated savannas, often in close juxtaposition, in eastern Africa between 17–10 Ma (Bonnefille, 2010; Linder, 2017). Thus, the Early Miocene in eastern Africa may have seen a patchwork of more arid vegetation permitting some passage of arid‐adapted plants and animals. The Middle Miocene cooling and drying, which followed the Neogene warmth climax at 17–15 Ma (Figure 5F; Zachos et al., 2001), triggered the rapid aridification of east Africa and continued into the Pliocene and Pleistocene (Flower and Kennett, 1994; Trauth et al., 2009). The concurrent uplift of the East African Plateau also significantly factored into this aridification (Sepulchre et al., 2006; Kaspar et al., 2010). The associated adaptive changes in vegetation and fauna in response to this aridification in eastern Africa are well established (Cerling et al., 1997; deMenocal; Bobe, 2006; Feakins et al., 2013; Linder and Verboom, 2015; Charles‐Dominique et al., 2016; Linder, 2017). These include the diversification of arid‐adapted CAM lineages (Gilman et al., 2023; Holtum, 2023; Sage et al., 2023) (Figure 5C, the shift from C_3_‐ to C_4_‐grass‐dominated savannas (Peppe et al., 2023) (Figure 5D), the diversification of large bovid mammals (Figure 5F), and concomitant increase in herbivore‐resistant spiny vegetation (Figure 5E). This time period highlights the first large‐scale corridor for arid plant migration through eastern Africa.

The earliest evidence for hyperarid conditions in Africa during the Neogene is seen in the Namib at 17–16 Ma, a region that had been more mesic and wooded prior in the Early Miocene (Senut et al., 2009; Hoetzel et al., 2015). This onset of desiccation in the Namib, likely due to the upwelling of cold waters associated with the formation of the Benguela Current (Siesser, 1980; Van Zinderen Bakker and Mercer, 1986), thus established the first (SW) pole of aridity seen in the AAC disjunction pattern. Throughout the Late Miocene and into the Pliocene–Pleistocene, periods of aridification were enhanced throughout southern Africa, but variation in amount of precipitation (Figure 1A), summer vs. winter rain climates, and topography led to complex patterns of aridity and vegetation types (Figure 1B) and drove the remarkable floristic endemism and diversity seen today in southern Africa (Goldblatt, 1978; Verboom et al., 2009; Linder, 2014; Linder and Verboom, 2015). This extreme aridification spread north in Africa, reaching the Sahara around 7 Ma and NE Africa in the early Pliocene (Senut et al., 2009), and thus established the second (NE) pole of aridity. The early‐Pliocene in NE Africa was the warmest period in the last 5 Myr with semiarid vegetation followed by a transition to more arid conditions and grasslands giving way to shrublands at 4.3 Ma (Liddy et al., 2016). The climate during the remainder of the Pliocene and Pleistocene in both arid poles of Africa exhibited declining temperatures and varied in aridity, but these fluctuations were marked by extreme aridity at 2.8, 1.7, and 1.0 Ma (deMenocal, 1995, 2004; Bobe, 2006; Berner et al., 2022).

### New perspectives and future studies on the AAC disjunction

A complex distributional pattern involving many unrelated, arid‐adapted plant taxa exists across southern, eastern, and northern Africa (Verdcourt, 1969; de Winter, 1971; Thulin, 1994; Jürgens, 1997). A more recent subset of this African Rand Flora, the African arid corridor group, exhibits a discontinuous distribution occurring in southern Africa and into NE Africa and extending into the Arabian Peninsula. It is noteworthy that two of the oldest AAC disjunct examples retain elements of more NW African distribution: *Neurada* also exhibits a disjunct NW area in addition to NE Africa; *Limeum* possesses some species with a Rand Flora distribution in addition to at least one SW–NE disjunct pair. A striking finding of this meta‐analysis involving 73 examples of this AAC disjunction pattern between NE and SW Africa is the association of the disjunction ages to the rise and intensification of aridification in the Middle to Late Miocene, especially the Pliocene, and through the Pleistocene (Figure 5). This association is not only to the climatic aridification and its accompanying temperature drop seen across Africa (Figure 5G), but it extends as well to the African rise of C_4_ grasses, CAM succulence, spiny vegetation, and bovid lineages (Figure 5), key features indicated by Arakaki et al. (20122) and Charles‐Dominique et al. (2016). In fact, three‐quarters of the AAC disjunctions (52, 76%) are timed to the last 6 Myr, with the largest 2‐Myr interval in number of disjunctions occurring in the Pliocene. The earliest date documented for the rise of these features that signal aridification in the African flora and fauna is 18–20 Ma (Arakaki et al., 2011; Charles‐Dominique et al., 2016). Only five of the 73 AAC disjunctions are timed before this date. Two of these disjunctions occur early in the radiation of the African succulent clade in *Euphorbia*. The other three involve small families (Limeaceae, Neuradaceae, Wellstediaceae) with few genera, two of which (Wellstediaceae, Limeaceae) are monogeneric. These three may well represent arid clades arising in the more mesic Paleogene, but which have experienced considerable extinction over the Neogene and now appear relictual. These examples of the AAC disjunction that presumably pre‐date aridification from the Middle Miocene are reminiscent of five fynbos clades in southern Africa (e.g., Restionaceae, Bruniaceae) that have been shown to substantially pre‐date the Middle Miocene rise of aridification giving rise to that specialized biome (Linder, 2005; Verboom et al., 2009; Linder and Verboom, 2015).

What are the processes that have given rise to this AAC disjunction? This question—*How do disjunctions form?*—is a fundamental issue that the field of historical biogeography attempts to resolve with its diverse toolkit comprising floristic and faunistic databases, phylogenetics, paleoclimatology, paleogeology, the fossil record, and a suite of analytical approaches once a chronogram is obtained. In the context of the African Rand Flora disjunction, and the AAC disjunction pattern in particular, two main paradigms have been invoked to answer the question in different ways (reviewed by Thulin, 1994; Sanmartín et al., 2010; Bellstedt et al., 2012; García‐Aloy et al., 2017; Rincón‐Barrado et al., 2024). The first is the vicariance hypothesis: Disjunct species or clades are the remnants of an older widespread flora that went partly extinct due to the rise of a new geological barrier (geographical vicariance) or environmental change (ecological vicariance). The second paradigm is the dispersal hypothesis: Disjunct species or clades are the result of separate migrations over land corridors or long‐distance dispersal (LDD) events over pre‐existing barriers to isolated geographical regions, followed by diversification in isolation. Two studies have examined Rand Flora plant disjuncts by more explicit modeling of speciation modes, spatial patterns, diversification trajectories, extinction rates, niche dynamics, and expected phylogenetic patterns (Sanmartín et al., 2010; Rincón‐Barrado et al., 2024). Both concluded that ecological vicariance and ecogeographical speciation (and climate driven extinction) explain the origin of the oldest disjunct clades (e.g., between NW Africa and either NE or southern Africa), but that ecological speciation and dispersal‐mediated speciation explain more recent disjuncts.

What does this AAC meta‐analysis suggest about the origin of the NE‐SW African disjunction? Due to the extensive nature of the analysis with over 73 disjunct examples, this study clearly lacks the modelling power inherent in the studies by Sanmartín et al. (2010) and Rincón‐Barrado et al. (2024). However, the meta‐analysis does indicate that a vicariant paradigm that posits widespread arid floras broken up by mesic or montane barriers is unlikely except for the oldest AAC examples noted earlier. The wide distribution of time splits from the Middle Miocene to the present of these disjunctions and the greater importance of aridification rather than few periodic returns to more mesic conditions in eastern Africa preclude simple vicariance explanations for arid adapted AAC examples. Second, the results of this AAC meta‐analysis point instead to a dispersal‐mediated speciation scenario as suggested by Sanmartín et al. (2010) and Rincón‐Barrado et al. (2024) for later Rand Flora disjuncts. The geological context of east Africa indicates that arid corridors appear to have been operational throughout this time period after the Late Miocene and enhanced during the last 6 Myr, and likely as Milankovitch‐driven, recurring, separate corridors (e.g., Schrire et al., 2009; Duesing et al., 2021).

Arid corridors have been argued to be operational for animals as well, including ground squirrels (Herron et al., 2005), springhares (Matthee and Robinson, 1997), gemsboks (Iyengar et al., 2006), lizards (Kissling et al., 2016), giraffes (O'Connor et al., 2019), and especially birds (Winterbottom, 1967; Freitag and Robinson, 1993; Guinevere et al., 2024). The AAC corridor has been argued to be the basis of the species pump hypothesis in birds, whereby much of the southern African avian diversity arose when species adapted to drier xeric habitats colonized southern Africa from northern Africa during the Pliocene–Pleistocene climatic fluctuations (Kaboth‐Bahr et al., 2021; Guinevere et al., 2024). Additional support for the dispersal–migration scenario is that many genera exhibit continuous distributions across the AAC (e.g., *Combretum*, *Terminalia*, *Acacia*) and as already listed by Verdcourt (1969) in the first paper describing the AAC disjunction. Many of the widespread species may be dispersed longer distances via elephants (Dudley, 2000) or as wind‐blown tumbleweeds (Van der Pijl, 1982). Verdcourt (1969, p. 142) argued that both widespread as well as disjunct genera, all with different dispersal mechanisms, would be expected with an arid corridor that “was never wide, nor open for long, nor indeed perhaps ever absolutely continuous.”

Thus, we argue that the dispersal‐mediated speciation paradigm better fits the time splits of the AAC plant examples. This dispersal hypothesis along intermittent corridors, rather than LDD between NE and SW Africa, is consistent with the results showing that about two‐thirds of these AAC examples have passive seed or fruit dispersal (not fleshy or without obvious animal dispersal features); indeed, Verdcourt (1969) already had mentioned that many of the strict endemics to these two regions appear to possess poor dispersal mechanisms. The steeper ecological gradients in NE Africa and SW Africa relative to eastern Africa appear to have subsequently fostered allopatric speciation in many of these AAC examples (e.g., Holstein and Renner, 2011). Interestingly, Sanmartín et al. (2010) conclude their discussion on the African Rand Flora and specifically on the African NE–SW disjunction by suggesting that future work should focus on testing asymmetric dispersal patterns (northward vs. southward migrations) by incorporating dated chronograms of many examples. Our analysis indeed demonstrates a strong asymmetry with 63% of the AAC clades exhibiting a northward, SW to NE Africa, migration pattern, despite the lower dispersibility modeled for SW African lineages vs. NW counterparts by Sanmartín et al. (2010). Perhaps not surprising is that this predominantly northward migration route matches the northward sweep of aridity starting in the Namib region during the Middle Miocene and culminating in the Horn of Africa during the Pliocene.

## CONCLUSIONS

The African arid corridor disjunct pattern involves both animal and now at least 73 plant examples. As part of the larger African Rand Flora disjunction pattern across arid Africa, the presence and width of the AAC is linked to past climatic and geological events in the Miocene and accelerating into the Pliocene and Pleistocene. Although old, relictual AAC disjuncts date to before the Miocene, a large majority occurred during the last 6 Myr with a peak in the Pliocene. The AAC disjunction spans across the major angiosperm clades but with concentrations in families recognized as possessing morphological and physiological adaptations to aridity. Although displaying a diversity of arid‐adapted growth forms, physiologies, and dispersal mechanisms, woody or succulent perennials and vines dominate as do plants with passive seed dispersal. Dispersal‐mediated speciation with extinction (rather than long‐distance dispersal or vicariance) and more often from SW to NE Africa appears to better explain the formation of the AAC disjunction pattern.

Phylogenetic relationships within *Sesamothamnus* (Pedaliaceae), one of the first recognized examples of this disjunction, are well supported by next‐generation sequencing of several hundred nuclear genes and the plastome. The crown diversification of the genus occurred in the Late Miocene and gave rise to two clades restricted to either NE or SW Africa, each of which then diversified and evolved their own suites of arid adapted features. These relationships are somewhat surprising based on previous morphological studies and indicate that the AAC disjunction pattern in *Sesamothamnus* is quite old compared to most other examples.

## AUTHOR CONTRIBUTIONS

J.Z. and K.J.S. conceived and undertook the project; J.P.R., R.K., B.T.D., E.M.L., and A.R.L. assisted with data collection; B.T.D., J.P.R., and K.J.S. generated the chronogram; J.Z., R.K., B.T.D., and K.J.S. analyzed the data; K.J.S. and J.Z. led the writing with later contributions from all authors.

## Supporting information


**Appendix S1**. Morphological data set for *Sesamothamnus* of three vegetative and four floral traits used in parsimony and ML analyses of ancestral character reconstruction (see Appendix S5).

**Appendix S2.** Bayesian tree of *Sesamothamnus* and outgroup genera in the Pedaliaceae based on nearly complete plastomes obtained with anchored hybrid enrichment.

**Appendix S3.** BEAST chronogram of *Sesamothamnus* and outgroup genera based on nuclear phylogenomic data (see Figure 4) but with nodal dates and 95% CIs provided.

**Appendix S4.** Penalized likelihood chronogram of *Sesamothamnus* and outgroup genera. Dates and ranges obtained by using the Pedaliaceae crown date and the upper and lower 95% CI dates from Rose et al. ( 2022).

**Appendix S5.** The evolution of seven vegetative and floral traits in *Sesamothamnus* (Pedaliaceae) with parsimony reconstruction.

## Data Availability

Nucleotide alignments, phylogenetic trees, BEAST2 files, and R scripts necessary to reproduce the analyses in this paper are deposited on GitHub: https://github.com/jrosaceae/Sesamothamnus_AAC. Raw reads have been deposited in the NCBI Sequence Read Archive (SRA) as BioProject PRJNA1419806 for new sequences and PRJNA1214378 for previously used sequences.
