# Supplementary material for: Evaluating the African arid corridor hypothesis: A meta‐analysis including the phylogenetic and biogeographical history of Sesamothamnus
Source: Am J Bot. 2026 Apr 22;113(5):e70192. doi: 10.1002/ajb2.70192 (PMC13206203; doi:10.1002/ajb2.70192)
Supplement: Supplementary file 1 — Appendix S1. Morphological data set for Sesamothamnus of three vegetative and four floral traits used in parsimony and ML analyses of ancestral character reconstruction (see Appendix S5). [file AJB2-113-e70192-s004.pdf]

Appendix S1. Morphological data set for Sesamothamnus of three vegetative and four floral traits used in parsimony and ML analyses of ancestral character reconstruction (see Appendix S5). Character coding for species of Sesamothamnus is based on Ihlenfeldt (2002, 2010). Outgroup taxa were collectively scored with the plesiomorphic state seen across the tribe Sesameae, sister to Sesamothamnus, to avoid spurious results due to low sampling across the family Pedaliaceae.

**species**	**habit**	**succulence**	**mucilage hairs**	**petal color**	**petal fringe**
Sesamothamnus guerichii S02	shrub	high	stellate	yellow	entire
Sesamothamnus guerichii S12	shrub	high	stellate	yellow	entire
Sesamothamnus guerichii S04	shrub	high	stellate	yellow	entire
Sesamothamnus guerichii S11	shrub	high	stellate	yellow	entire
Sesamothamnus lugardii S14	shrub	medium	stellate	cream-yellow	entire
Sesamothamnus lugardii S10	shrub	medium	stellate	cream-yellow	entire
Sesamothamnus benguellensis	herb	high	stellate	cream-yellow	entire
Sesamothamnus leistneri	tree	high	quadrangular	cream-yellow	entire
Sesamothamnus rivae S07	small tree	low	quadrangular	white	entire
Sesamothamnus rivae S08	small tree	low	quadrangular	white	entire
Sesamothamnus busseanus	small tree	low	quadrangular	white	lacerated
Sesamum trilobum	herb	absent	quadrangular	non-white	entire
Uncarina abbreviata	herb	absent	quadrangular	non-white	entire
Uncarina platycarpa	herb	absent	quadrangular	non-white	entire
Pterodiscus aurantiacus	herb	absent	quadrangular	non-white	entire

	**petal spur**	**anther position**
Sesamothamnus guerichii S02	sac-like	inserted
Sesamothamnus guerichii S12	sac-like	inserted
Sesamothamnus guerichii S04	sac-like	inserted
Sesamothamnus guerichii S11	sac-like	inserted
Sesamothamnus lugardii S14	long	exserted
Sesamothamnus lugardii S10	long	exserted
Sesamothamnus benguellensis	long	exserted
Sesamothamnus leistneri	absemt	exserted
Sesamothamnus rivae S07	long	exserted
Sesamothamnus rivae S08	long	exserted
Sesamothamnus busseanus	long	exserted
Sesamum trilobum	absent	exserted
Uncarina abbreviata	absent	exserted
Uncarina platycarpa	absent	exserted
Pterodiscus aurantiacus	absent	exserted
