# Supplementary material for: Evaluating the African arid corridor hypothesis: A meta‐analysis including the phylogenetic and biogeographical history of Sesamothamnus
Source: Am J Bot. 2026 Apr 22;113(5):e70192. doi: 10.1002/ajb2.70192 (PMC13206203; doi:10.1002/ajb2.70192)

Appendix S2. Bayesian tree of *Sesamothamnus* and outgroup genera in the Pedaliaceae based on nearly complete plastomes obtained with anchored hybrid enrichment (AHE). Posterior probability values are indicated. Due to the long branch of *Pterodiscus* , its branch length is shown at 50% scale. This topology is the same as the ASTRAL tree (Fig. 3) based on 512 nuclear genes with AHE. The RAxML bootstrap topology based on plastomes is identical except that *S. guerichii* S02 is sister to a polytomy of the remaining three accessions of *S. guerichii* with all nodes in the tree supported at 100%. The distributions of Sesamothamnus species are color-coded to match placements on the tree.

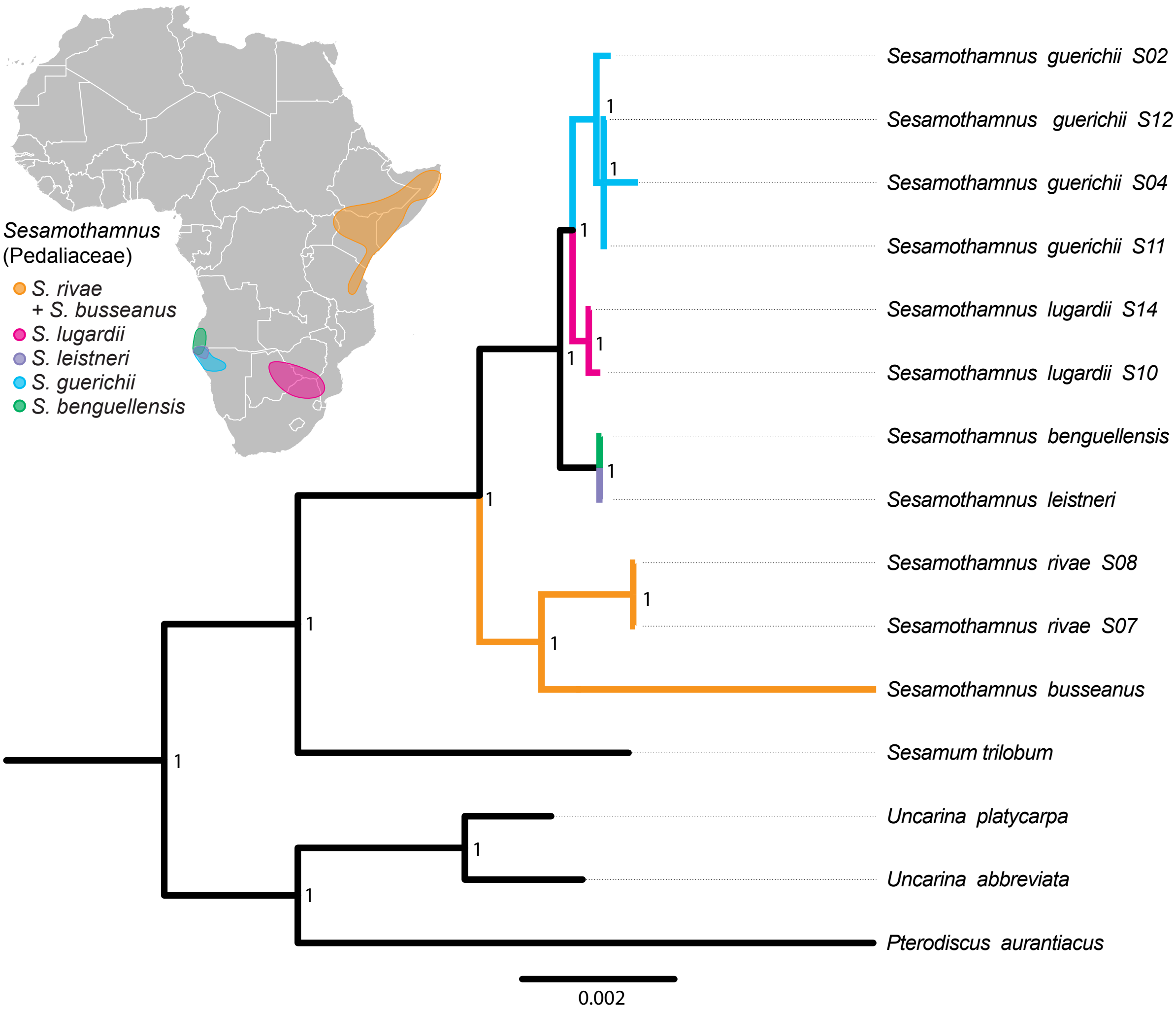

Supplement: Supplementary file 2 — Appendix S2. Bayesian tree of Sesamothamnus and outgroup genera in the Pedaliaceae based on nearly complete plastomes obtained with anchored hybrid enrichment. [file AJB2-113-e70192-s003.pdf]
