# Supplementary material for: Evaluating the African arid corridor hypothesis: A meta‐analysis including the phylogenetic and biogeographical history of Sesamothamnus
Source: Am J Bot. 2026 Apr 22;113(5):e70192. doi: 10.1002/ajb2.70192 (PMC13206203; doi:10.1002/ajb2.70192)

Appendix S3. BEAST chronogram of *Sesamonthamnus* and outgroup genera based on nuclear phylogenomic data (see Fig. 4) but with nodal dates and 95% CIs provided.

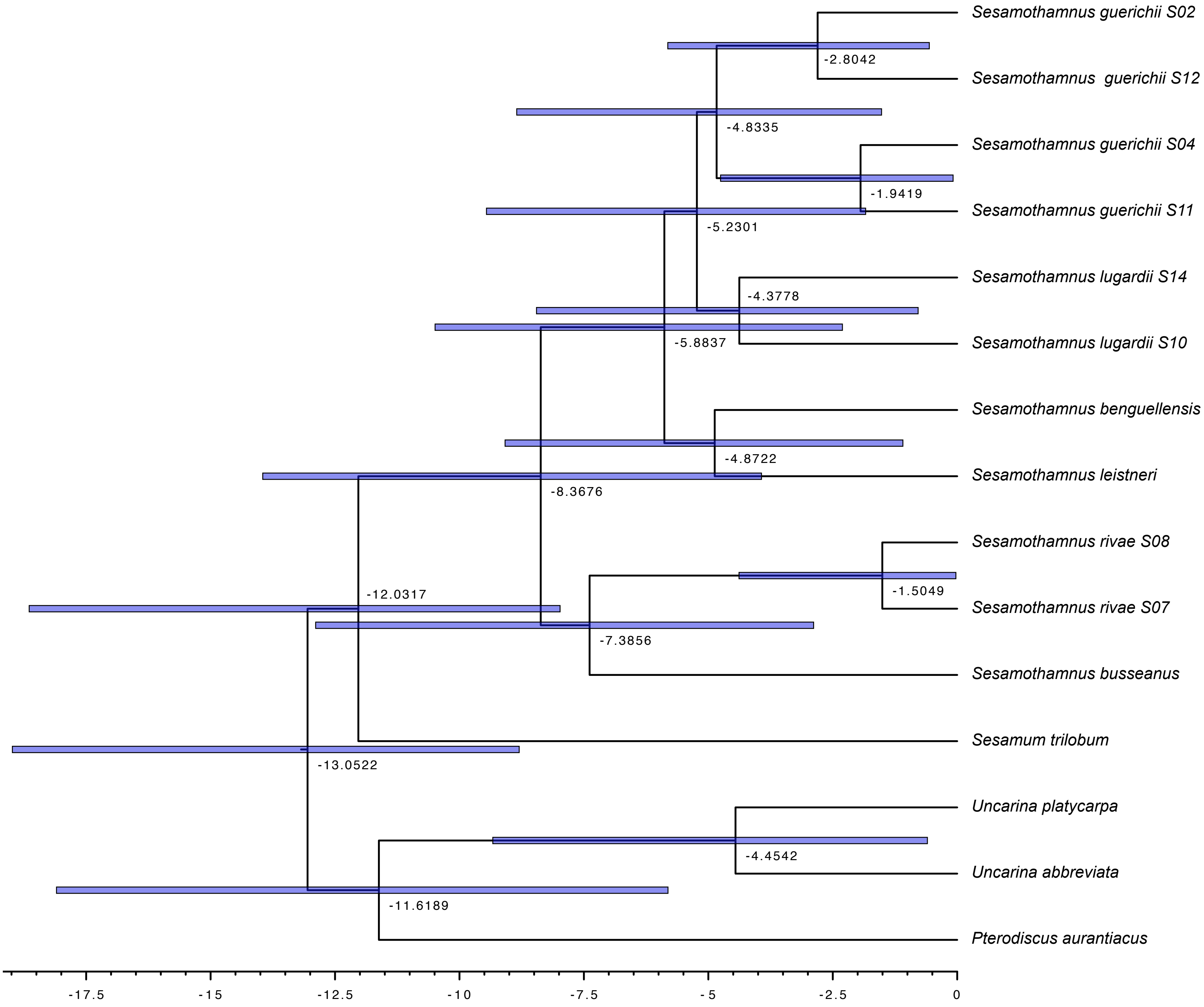

Supplement: Supplementary file 3 — Appendix S3. BEAST chronogram of Sesamothamnus and outgroup genera based on nuclear phylogenomic data (see Figure 4) but with nodal dates and 95% CIs provided. [file AJB2-113-e70192-s005.pdf]
