# Supplementary material for: Evaluating the African arid corridor hypothesis: A meta‐analysis including the phylogenetic and biogeographical history of Sesamothamnus
Source: Am J Bot. 2026 Apr 22;113(5):e70192. doi: 10.1002/ajb2.70192 (PMC13206203; doi:10.1002/ajb2.70192)

Appendix S4. Penalized likelihood chronogram of ***Sesamothamnus*** and outgroup genera. Dates and ranges obtained by using the Pedaliaceae crown date and the upper and lower 95% CI dates from Rose et al. (2022).

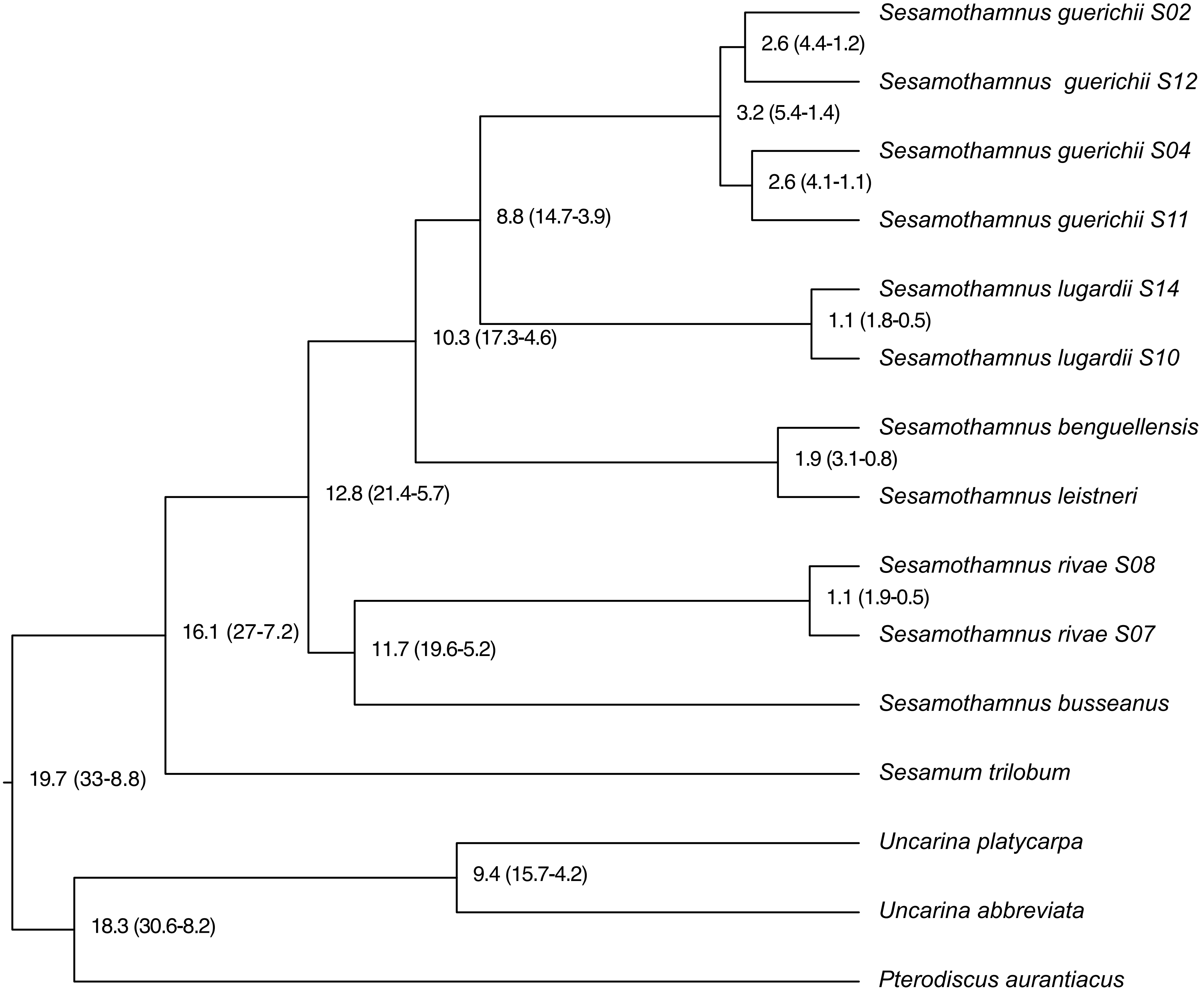

Supplement: Supplementary file 4 — Appendix S4. Penalized likelihood chronogram of Sesamothamnus and outgroup genera. Dates and ranges obtained by using the Pedaliaceae crown date and the upper and lower 95% CI dates from Rose et al. ( 2022). [file AJB2-113-e70192-s002.pdf]
