# Supplementary material for: Evaluating the African arid corridor hypothesis: A meta‐analysis including the phylogenetic and biogeographical history of Sesamothamnus
Source: Am J Bot. 2026 Apr 22;113(5):e70192. doi: 10.1002/ajb2.70192 (PMC13206203; doi:10.1002/ajb2.70192)

**Appendix S5.** The evolution of seven vegetative and floral traits in *Sesamothamnus* (Pedaliaceae) with parsimony reconstruction. The most parsimonious reconstruction (MPR) and the accelerated transformation (ACCTRAN) reconstruction are presented. Trait evolution is summarized on the BEAST chronogram in Fig. 4. Traits are scored as done by Ihlenfeldt (2002, 2010) for *Sesamothamnus* with all outgroups given the plesiomorphic states as seen in the sister group, the tribe Sesameae (see Appendix S1).

# Contents

**Habit** 2
MPR . . . . . 2
ACCTRAN . . . . . 2

**Succulence** 3
MPR . . . . . 3
ACCTRAN . . . . . 3

**Mucilage** 4
MPR . . . . . 4
ACCTRAN . . . . . 4

**Flower Color** 5
MPR . . . . . 5
ACCTRAN . . . . . 5

**Petal fringe** 6
MPR . . . . . 6
ACCTRAN . . . . . 6

**Spur** 7
MPR . . . . . 7
ACCTRAN . . . . . 7

**Anther position** 8
MPR . . . . . 8
ACCTRAN . . . . . 8

# Habit

## MPR

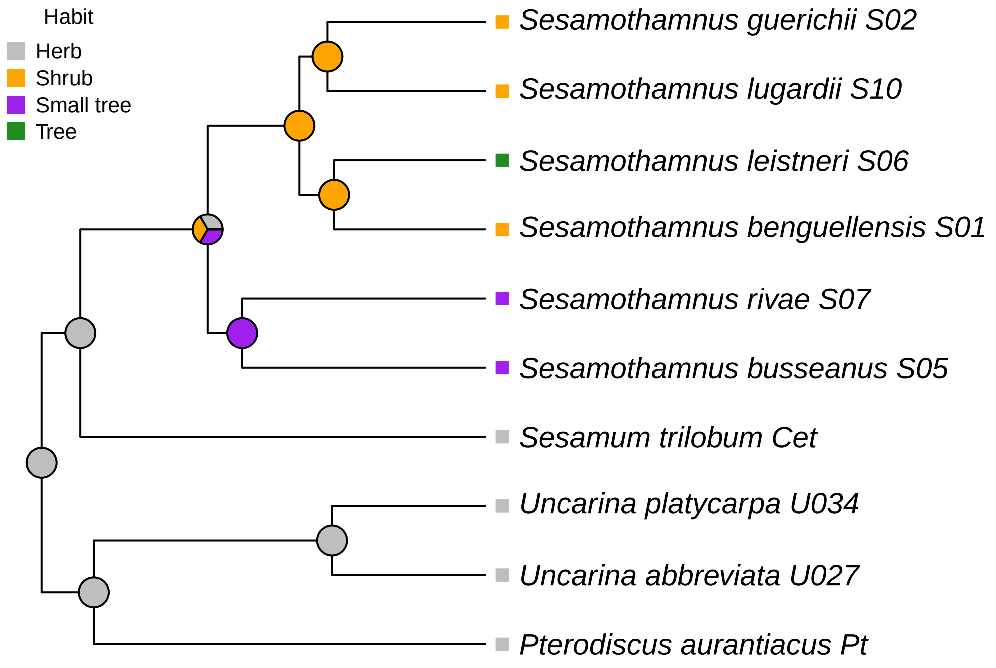


## ACCTRAN

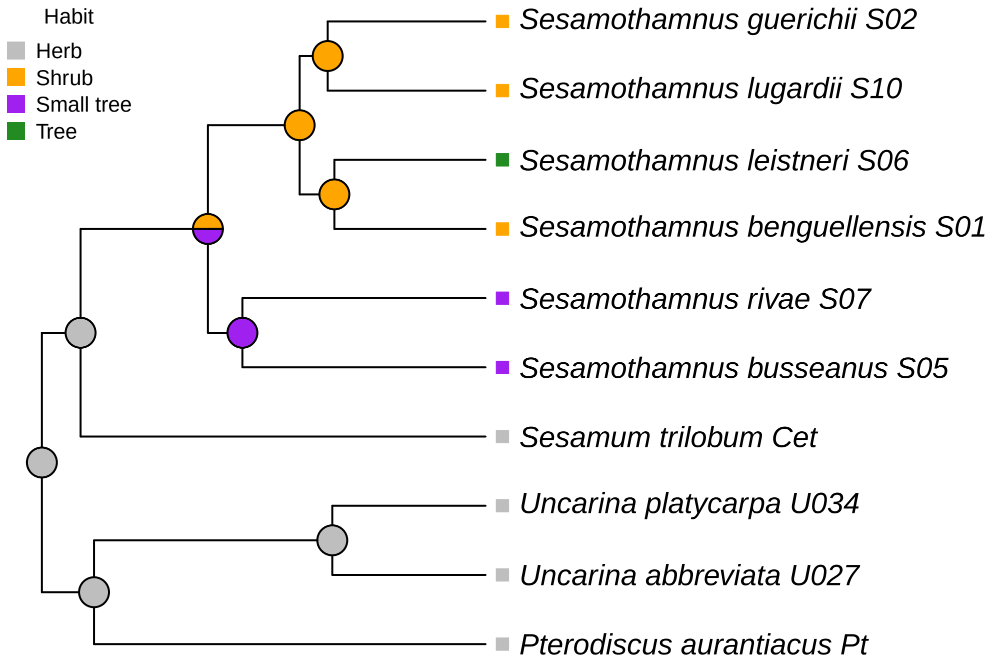

# Succulence

## MPR

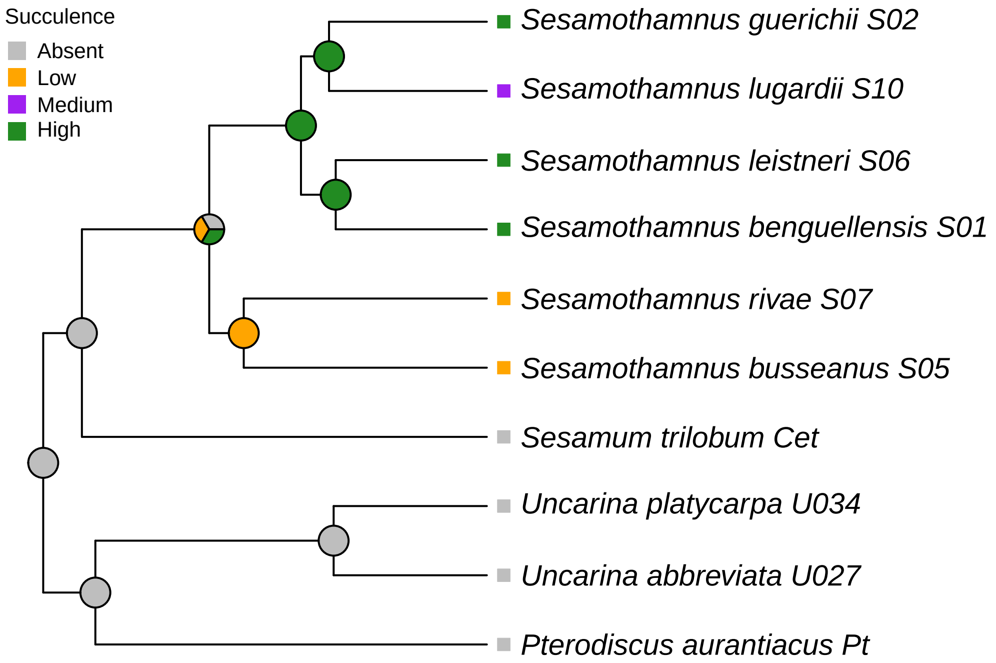


## ACCTRAN

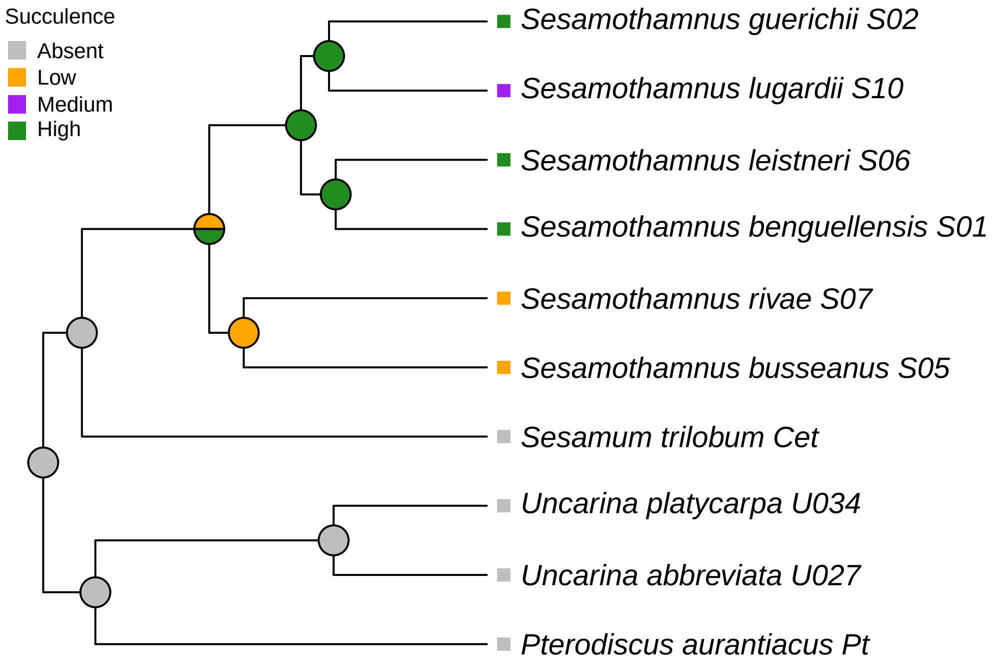

# Mucilage

## MPR

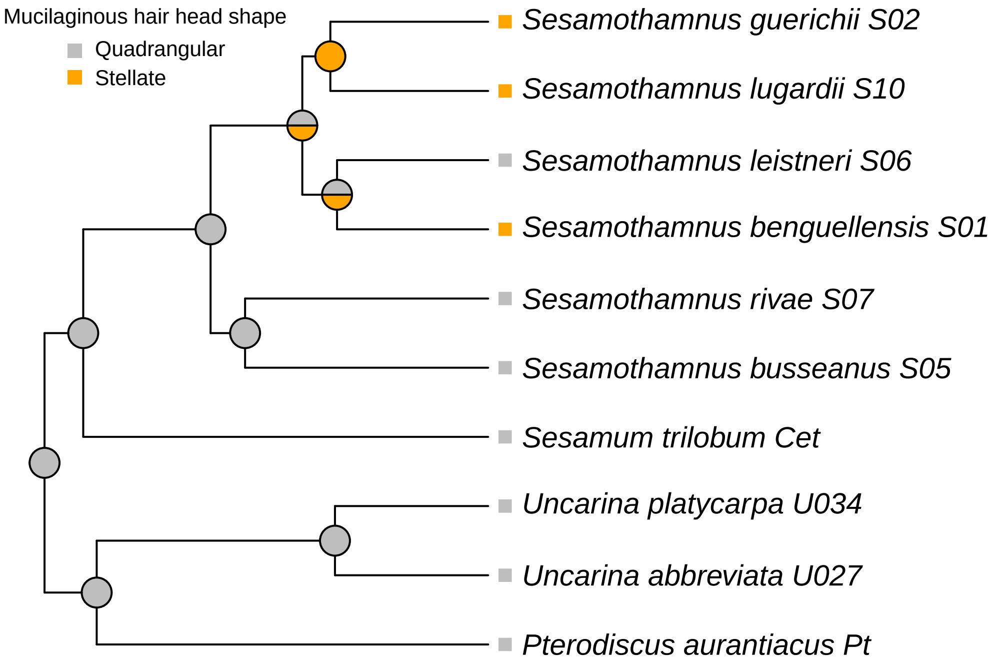


## ACCTRAN

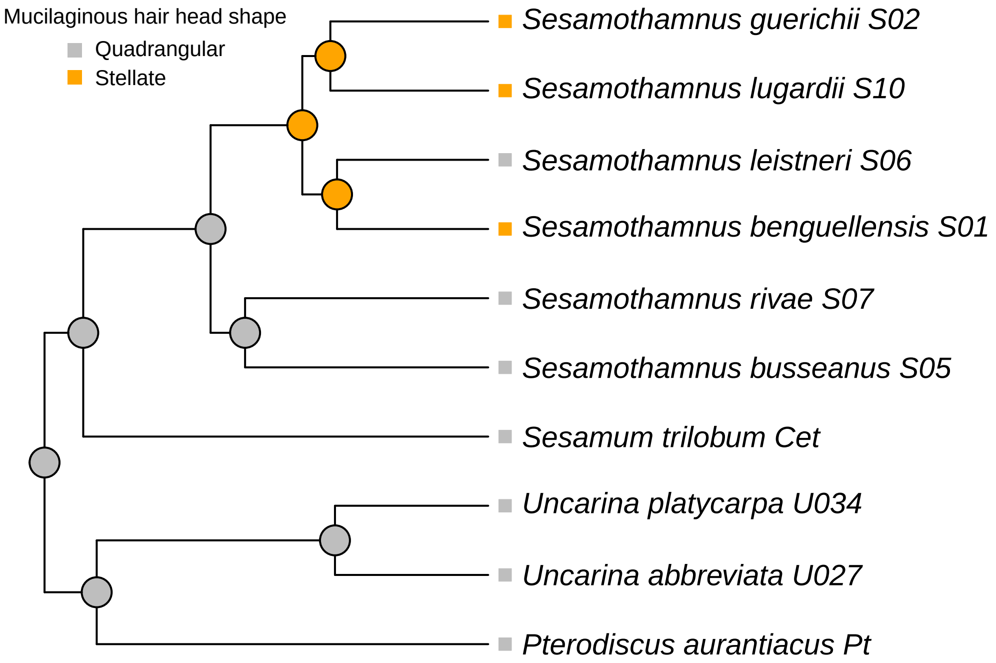

# Flower Color

## MPR

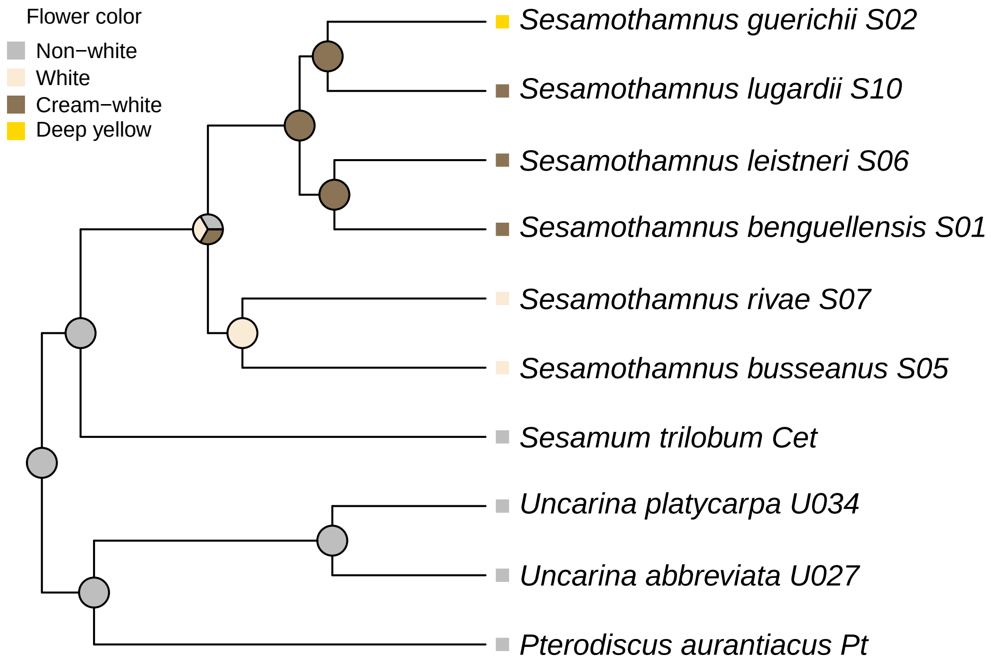


## ACCTRAN

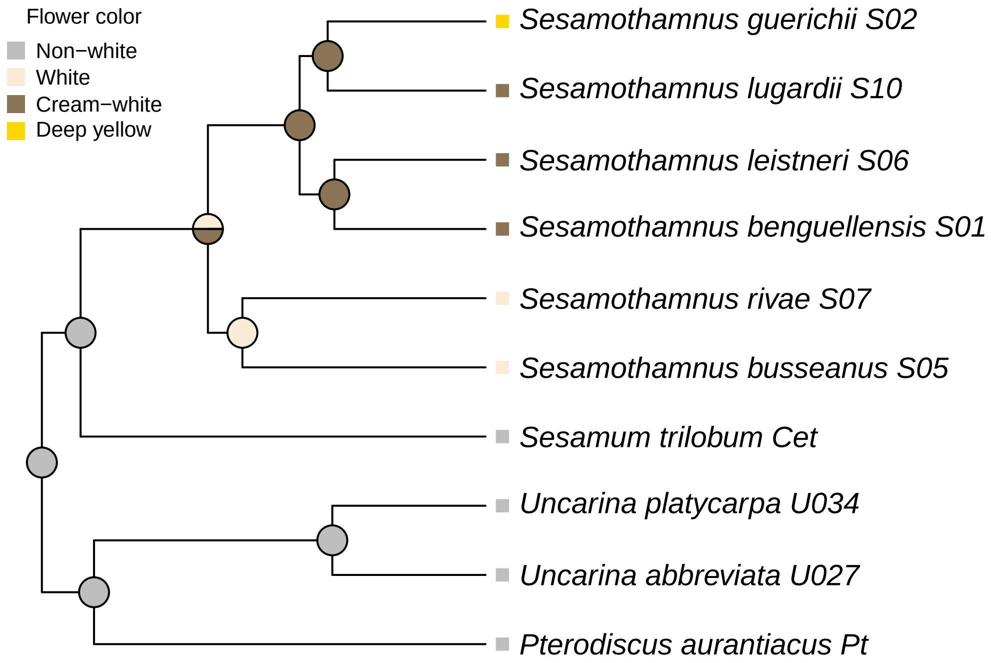

# Petal fringe

## MPR

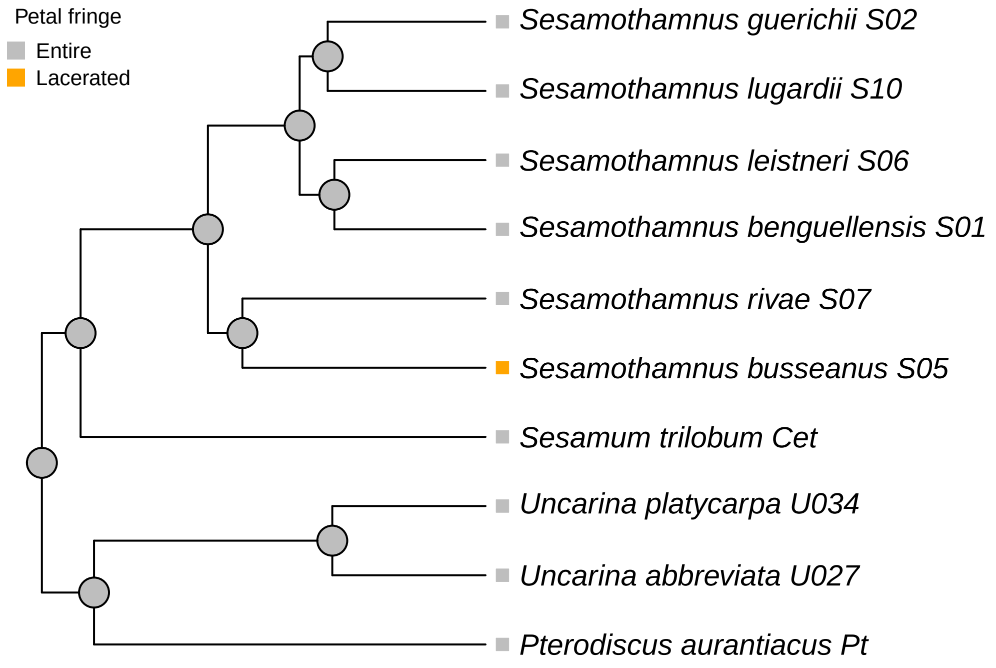


## ACCTRAN

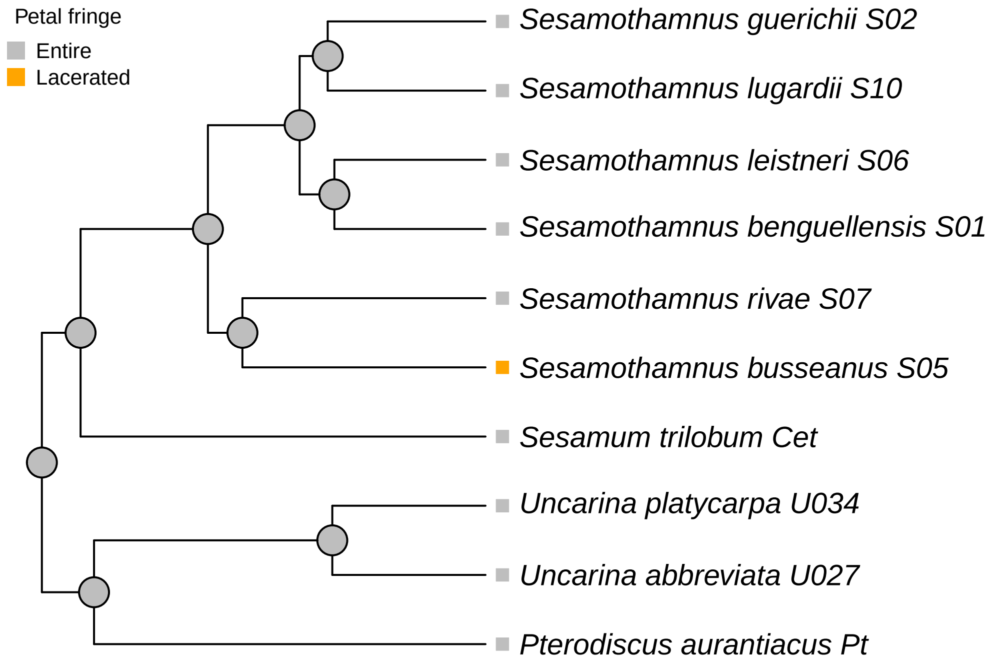

# Spur

## MPR

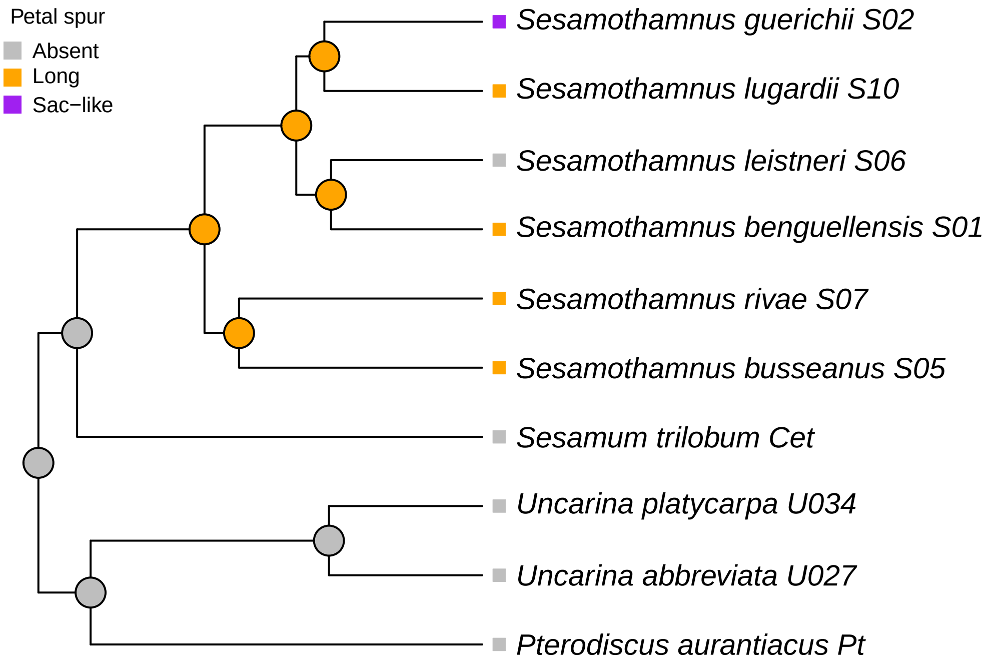


## ACCTRAN

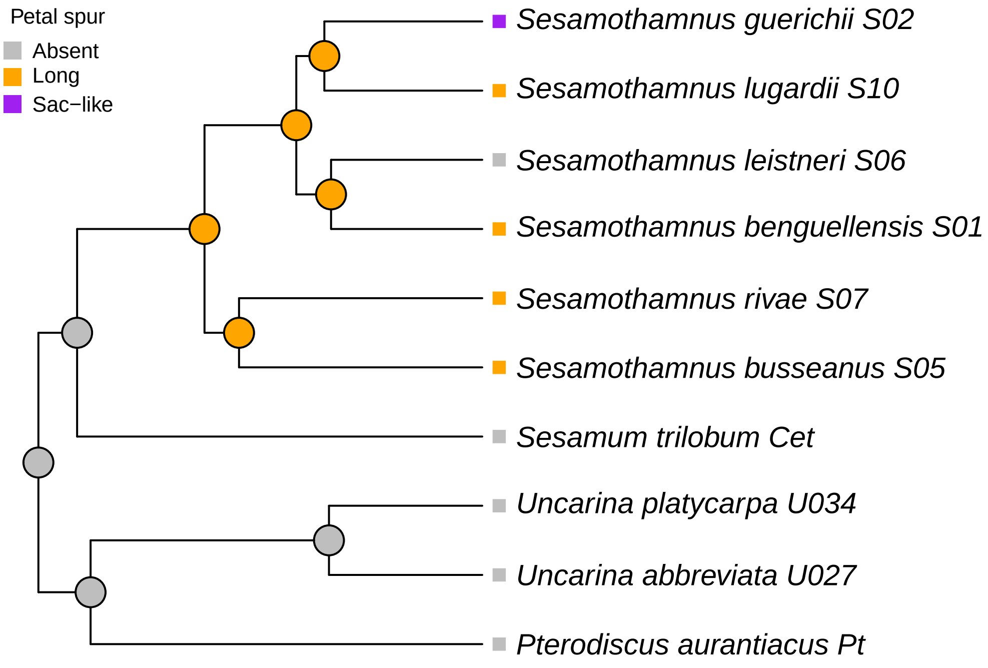

# Anther position

## MPR

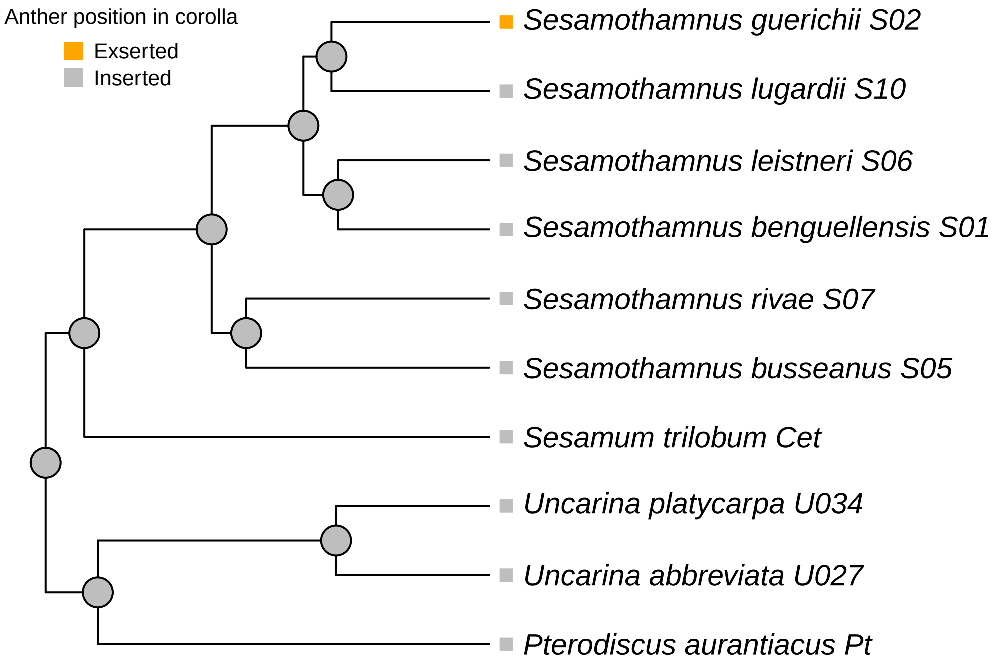


## ACCTRAN

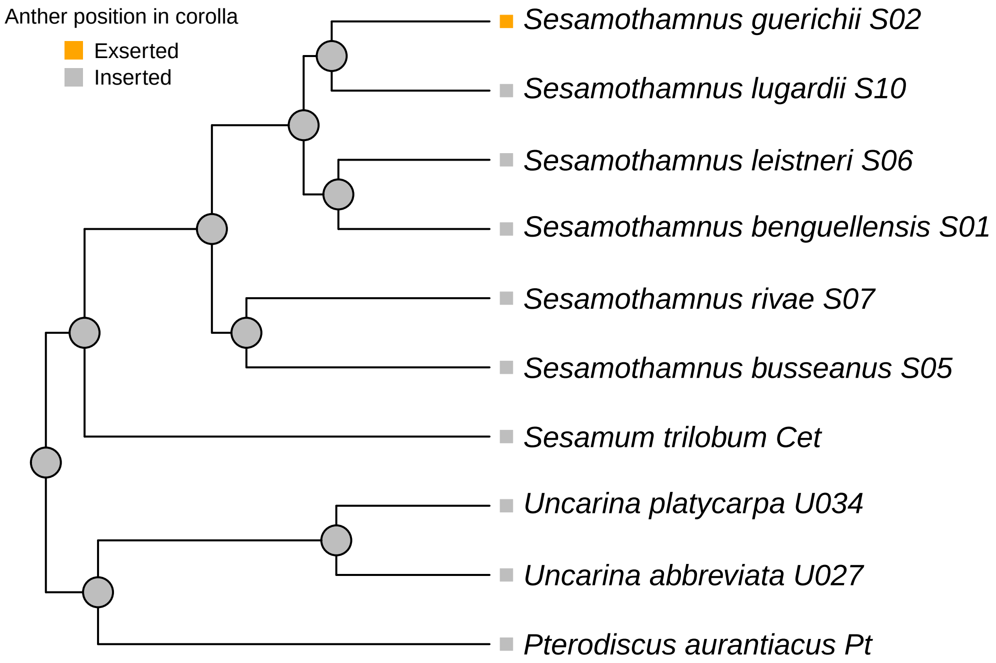

Supplement: Supplementary file 5 — Appendix S5. The evolution of seven vegetative and floral traits in Sesamothamnus (Pedaliaceae) with parsimony reconstruction. [file AJB2-113-e70192-s001.pdf]
